# Supplementary material for: Mouse models of Japanese encephalitis virus infection: A systematic review and meta-analysis using a meta-regression approach
Source: PLoS Negl Trop Dis. 2022 Feb 10;16(2):e0010116. doi: 10.1371/journal.pntd.0010116 (PMC8865681; doi:10.1371/journal.pntd.0010116)
Supplement: S1 Data Protocol — (DOCX) [file pntd.0010116.s001.docx]

**S1 Data Protocol: A systematic review of mouse models of *Japanese encephalitis*.**

**Background**

There have been reports published of experiments using *Japanese encephalitis virus* (JEV) in mouse models dating back to the 1930s, ever since JEV was first isolated in 1935. These models have been used to address a wide variety of different questions, such as the role of various components of the immune system in protection from JEV and some fundamentals of pathology and pathogenesis. One conclusion that has been drawn from these studies, supported by parallel observations in humans and other viruses, is that neutralizing antibody is protective against disease caused by JEV in mice.

In recent years there has been a focus on the quality of experimental design and reporting of animal models, with a view to improving the process of pre-clinical development of therapeutics. Several fields have produced disappointing results when attempting to translate interventions to clinical practice, bringing renewed focus on the process of generating important pre-clinical data. There are currently no effective treatments for JE, and although there have effective vaccines since at least the 1950s still 80% of cases occur in areas with established vaccination programs. Whatever their relative merits, animal data are likely to be used to inform the development of any new treatments for JE. In the last 5-10 years many of the high impact publications in JE have used animal models, with several new key insights. However, animal models of JE are highly variable and the literature can be confusing. Therefore, a systematic review of mice models of JE is timely, with a view to understanding which characteristics of models are the most important, to aid with consistency between laboratories and to ensure homogeneous reporting of results.

**Research question**

Primary question: What is the performance of existing mouse models of JEV infection? This will involve a critical appraisal of the quality of reporting, as well as the validity and reproducibility of the models.

Secondary questions:

1. Does virus dose correlate with mortality?
2. Does mouse age inversely correlate with mortality?
3. Does mouse strain affect mortality?
4. Does viral strain correlate with mortality?
5. Does the route of inoculation affect mortality?
6. Does the laboratory affect mortality?
7. Does publication year affect mortality?

**Specific aims**

1. Summary of quality of reporting of existing mouse models of JEV infection
2. To test the relationship of virus strain, dose, route of administration, mouse strain and age on mortality from JEV infection of mice.
3. To develop guidance on the set up and reporting of mouse models of JE

**Search strategy**

Pubmed will be searched using the terms ("encephalitis, Japanese"[MeSH Terms] OR ("encephalitis"[All Fields] AND "Japanese"[All Fields]) OR "Japanese encephalitis"[All Fields] OR ("Japanese"[All Fields] AND "encephalitis"[All Fields]) OR "je"[All Fields] OR "jev"[All Fields]) AND ("mice"[MeSH Terms] OR "mice"[All Fields] OR "mouse"[All Fields] OR "mice"[MeSH Terms] OR "mice"[All Fields] OR mus[All Fields]). The search date range will be 1935 (the year of first isolation of JEV) to August 2020. We will filter the search to those including abstracts in English. Retrieved references will be downloaded into EndNote for the removal of duplicate studies, which will also be checked manually.

Abstracts will be screened according to the criteria above; ambiguity will be resolved by reading the full text. Publications will be screened by one author and categorized into “included (for analysis),” “included (for narrative review only),” “excluded” and “uncertain.” The “uncertain” category will be resolved by another author independently.

Publications will be screened by a single author, with a subset of 10% checked for consistency by a second author.

**Inclusion criteria**

Any publication that includes an experiment meeting the following criteria will be included:

1. JEV is inoculated into mice

2. Virus dose is reported

3. JEV strain or source is reported

4. Mouse strain is reported

6. Mortality is reported, either death or humane endpoint (primary analysis & outcome measures, secondary outcome measures 1 and 2) or another pathological outcome is reported (secondary outcome 3)

6. Publication in English

Age of the mice at use will also be included, however non-reporting of age will not serve as an exclusion from the primary analysis.

Publications such as reviews or re-analyses of existing data will not be included unless they provide insight into an existing paper (such as a more detailed breakdown of mortality), in such cases data will be concatenated into one study. Any publication reporting primary data can be included.

**Exclusion criteria**

Inoculation using non-pathogenic JEV (for example the vaccine strain SA14-14-2)

Data on individual animals not reported, and it is not possible to extract the data.

Publications meeting these criteria will not be included in the primary analysis. However, publications using non-pathogenic JEV can still be included if they use virulent JEV inoculations in other groups of mice, for example as a challenge model, or if reversion to wild type phenotype is described. Studies not reporting mortality, for example because mice are sacrificed before death for pathology or mechanistic studies, or not reporting individual animal level data, will be counted and presented in the Prisma flow diagram.

**Data extraction**

The following data will be extracted:

PMID

Year of publication

Senior author

Institution/laboratory

Country

Mouse strain

Mouse gender

Mouse age

Virus strain

Genotype

Dose of virus/units

Route of administration

Number of mice (total, surviving, dying)

Survival time

The laboratory will be identified based either on the affiliations of the authors or from the ethics statement, making the assumption that the institution undertaking ethical review of the experiments is also the one holding the animals.

Data will be extracted by a single author. A subset of 10% of included publications will be cross-checked by another author.

**Analysis plan**

- 1. Quality assessment

The quality of studies will be assessed based on ten standard quality measures used previously for animal model meta-analyses in the CAMARADES database:

1. Peer reviewed publication
2. Statement that experimental temperature was controlled
3. Random allocation to experimental group
4. Treatment blinded to investigator
5. Outcome blinded to analyzing investigator
6. Statement whether neuroprotective anesthesia used
7. Appropriate model used
8. Statement that sample size calculation was done
9. Statement that relevant animal welfare regulations were followed (this is also used to infer where the experiments were done)
10. Conflict of interest statement given

In addition, the following will be extracted as study quality measures:

1. Statement of the intent of the experiments conducted
2. Mouse strain used stated
3. Virus used stated
4. Dose of virus given
5. Route of inoculation given
6. Age of mice at inoculation explicitly stated or easily calculated (e.g. “mice were immunized at 6 weeks of age ….. and challenged 2 weeks after immunization”)

Each study will be allocated a score out of 16 based on these quality measures.

- 1. Data analysis

Several factors can be foreseen which may confound the analysis. These are:

*Reporting of dose of virus*

Although the ideal reporting of virus dose is in directly quantified infectious particles, or plaque forming units (PFU), many papers do not use this measure and instead report the tissue culture infectious dose required to infect 50% of a panel of cell monolayers (TCID_50_) or a multiple of the dose required to kill 50% of infected mice (lethal dose_50_, LD_50_). Genome copies measured by RT-qPCR may also serve as a measure of viral inoculum, though in practice this is rarely used. In publications where a quantitative measure (PFU or TCID_50_) is used this will always be the variable used in analysis. TCID_50_ will be converted to PFU using the formula PFU = 0.7 x TCID_50_. Models quoting solely the LD_50_ or multiple thereof will be analyzed separately.

*Strain of JEV*

Many different strains of JEV have been used in the laboratory since the first isolation of JEV. Some of these have become adapted to laboratory conditions over many years and may not exhibit the same pathogenic properties as when first isolated. For this reason, time between experimental reports, measured in years, will be investigated as a confounding variable. In cases where strains of JEV are known to be the same although nomenclature may have changed over time, they will be analyzed both together and separately. Non-pathogenic JEV strains will not be included, but there are some strains that show reduced pathogenicity (e.g SA14). These will be included in exploratory analyses where they are analyzed separately to other viral strains.

*Primary analyses*

Our null hypotheses are that there is no relationship between viral dose, strain, mouse strain, route of inoculation, age and publication year on mortality of mice after inoculation with JEV. Variables of interest will be plotted against mortality at the level of individual experiments. Publication bias will be explored as appropriate using Funnel or Galbraith plots. Individual and aggregated forest plots will be used to summarize data, stratified by key variables. Meta-regression using a percentage scale for the outcome will be used to quantify the impact of experiment-level covariates on heterogeneity of outcomes. The generalized R2 and I2 statistics, likelihood ratio test and the AIC will be used to judge model fit and routine regression diagnostics to test model assumptions. Should any studies be identified as outliers using this strategy, primary analyses will be repeated omitting the outlying studies. A multivariable analysis will be performed using all of the variables above in a single model. Sensitivity analyses will consider the effects of the strength of effect of each variable individually by assessing the effect of leaving them out of the model on the AIC.
