# Supplementary material for: Mouse models of Japanese encephalitis virus infection: A systematic review and meta-analysis using a meta-regression approach
Source: PLoS Negl Trop Dis. 2022 Feb 10;16(2):e0010116. doi: 10.1371/journal.pntd.0010116 (PMC8865681; doi:10.1371/journal.pntd.0010116)
Supplement: S1 Data — (DOCX) [file pntd.0010116.s002.docx]

**S1 Data: R code for meta-regression analysis**

**1) Effect of mouse strain as a moderator in the meta-regression model**

> summary(metareg.mouse_strain< rma(measure="PLO",xi=no_died,ni=no_mice,mods=~mouse_strain_2-1,data=jemouse,method="DL"))

Mixed-Effects Model (k = 487; tau^2 estimator: DL)

logLik deviance AIC BIC AICc

-988.0088 969.2381 2006.0177 2068.8416 2007.0368

tau^2 (estimated amount of residual heterogeneity): 1.5016 (SE = 0.2028)

tau (square root of estimated tau^2 value): 1.2254

I^2 (residual heterogeneity / unaccounted variability): 63.60%

H^2 (unaccounted variability / sampling variability): 2.75

Test for Residual Heterogeneity:

QE(df = 473) = 1299.4890, p-val < .0001

Test of Moderators (coefficients 1:14):

QM(df = 14) = 242.0741, p-val < .0001

Model Results:

estimate se zval pval ci.lb ci.ub

mouse_strain_2C57BL/6 0.0138 0.1366 0.1010 0.9196 -0.2539 0.2815

mouse_strain_2BALB/c 1.3372 0.1486 8.9969 <.0001 1.0459 1.6285 ***

mouse_strain_2Swiss 1.8820 0.2520 7.4676 <.0001 1.3881 2.3760 ***

mouse_strain_2C3H/He 0.7711 0.2256 3.4184 0.0006 0.3290 1.2132 ***

mouse_strain_2ICR 2.0158 0.3020 6.6749 <.0001 1.4239 2.6077 ***

mouse_strain_2ddY -0.6414 0.2901 -2.2106 0.0271 -1.2101 -0.0727 *

mouse_strain_2129 -1.2289 0.6459 -1.9026 0.0571 -2.4948 0.0370 .

mouse_strain_2AA -2.4620 0.6061 -4.0622 <.0001 -3.6499 -1.2741 ***

mouse_strain_2KK -1.8780 0.5721 -3.2827 0.0010 -2.9993 -0.7567 **

mouse_strain_2NC -1.5043 0.5994 -2.5095 0.0121 -2.6791 -0.3294 *

mouse_strain_2RR -0.9235 0.6108 -1.5118 0.1306 -2.1207 0.2737

mouse_strain_2DBA/2 -0.4055 1.3077 -0.3101 0.7565 -2.9684 2.1575

mouse_strain_2CF1 2.2981 1.3608 1.6888 0.0913 -0.3690 4.9652 .

mouse_strain_2FVB/J 2.5649 1.9119 1.3415 0.1797 -1.1824 6.3123

---

Signif. codes: 0 ‘***’ 0.001 ‘**’ 0.01 ‘*’ 0.05 ‘.’ 0.1 ‘ ’ 1

> anova(metareg.base,metareg.mouse_strain)

df AIC BIC AICc logLik LRT pval QE tau^2 R^2

Full 15 2006.0177 2068.8416 2007.0368 -988.0088 1299.4890 1.5016

Reduced 2 2141.8600 2150.2366 2141.8848 -1068.9300 161.8424 <.0001 1624.2610 1.9904 24.5555%

**2) Effect of mouse age as a moderator in the meta-regression model**

> summary(metareg.mouse_age<-rma(measure="PLO",xi=no_died,ni=no_mice,mods=~mouse_age_mths-1,data=jemouse[!is.na(jemouse$mouse_age_mths),],method="DL"))

Mixed-Effects Model (k = 482; tau^2 estimator: DL)

logLik deviance AIC BIC AICc

-999.6002 998.8273 2009.2005 2030.0902 2009.3265

tau^2 (estimated amount of residual heterogeneity): 1.6324 (SE = 0.2142)

tau (square root of estimated tau^2 value): 1.2777

I^2 (residual heterogeneity / unaccounted variability): 65.19%

H^2 (unaccounted variability / sampling variability): 2.87

Test for Residual Heterogeneity:

QE(df = 478) = 1373.1837, p-val < .0001

Test of Moderators (coefficients 1:4):

QM(df = 4) = 188.9086, p-val < .0001

Model Results:

estimate se zval pval ci.lb ci.ub

mouse_age_mths1 1.2253 0.1167 10.4996 <.0001 0.9966 1.4541 ***

mouse_age_mths2 0.6721 0.1416 4.7470 <.0001 0.3946 0.9496 ***

mouse_age_mths3 0.9988 0.3573 2.7957 0.0052 0.2986 1.6990 **

mouse_age_mths4 -1.2572 0.1809 -6.9510 <.0001 -1.6117 -0.9027 ***

---

Signif. codes: 0 ‘***’ 0.001 ‘**’ 0.01 ‘*’ 0.05 ‘.’ 0.1 ‘ ’ 1

> summary(metareg.empty.base<-rma(measure="PLO",xi=no_died,ni=no_mice,data=jemouse[!is.na(jemouse$mouse_age_mths),],method="DL"))

Random-Effects Model (k = 482; tau^2 estimator: DL)

logLik deviance AIC BIC AICc

-1059.8718 1119.3706 2123.7437 2132.0996 2123.7687

tau^2 (estimated amount of total heterogeneity): 2.0071 (SE = 0.2574)

tau (square root of estimated tau^2 value): 1.4167

I^2 (total heterogeneity / total variability): 70.04%

H^2 (total variability / sampling variability): 3.34

Test for Heterogeneity:

Q(df = 481) = 1605.3058, p-val < .0001

Model Results:

estimate se zval pval ci.lb ci.ub

0.5910 0.0838 7.0551 <.0001 0.4268 0.7552 ***

---

Signif. codes: 0 ‘***’ 0.001 ‘**’ 0.01 ‘*’ 0.05 ‘.’ 0.1 ‘ ’ 1

> anova(metareg.empty.base,metareg.mouse_age)

df AIC BIC AICc logLik LRT pval QE tau^2 R^2

Full 5 2009.2005 2030.0902 2009.3265 -999.6002 1373.1837 1.6324

Reduced 2 2123.7437 2132.0996 2123.7687 -1059.8718 120.5432 <.0001 1605.3058 2.0071 18.6673%

**3) Effect of mouse sex as a moderator in the meta-regression model**

> summary(metareg.mouse_sex<-rma(measure="PLO",xi=no_died,ni=no_mice,mods=~mouse_sex-1,data=jemouse,method="DL"))

Mixed-Effects Model (k = 487; tau^2 estimator: DL)

logLik deviance AIC BIC AICc

-1056.7946 1106.8095 2121.5892 2138.3422 2121.6721

tau^2 (estimated amount of residual heterogeneity): 1.8683 (SE = 0.2392)

tau (square root of estimated tau^2 value): 1.3668

I^2 (residual heterogeneity / unaccounted variability): 68.66%

H^2 (unaccounted variability / sampling variability): 3.19

Test for Residual Heterogeneity:

QE(df = 484) = 1544.5557, p-val < .0001

Test of Moderators (coefficients 1:3):

QM(df = 3) = 82.5307, p-val < .0001

Model Results:

estimate se zval pval ci.lb ci.ub

mouse_sexFemale 1.0478 0.1175 8.9159 <.0001 0.8174 1.2781 ***

mouse_sexMale 0.5477 0.6687 0.8191 0.4128 -0.7630 1.8584

mouse_sexNA 0.1761 0.1144 1.5386 0.1239 -0.0482 0.4004

---

Signif. codes: 0 ‘***’ 0.001 ‘**’ 0.01 ‘*’ 0.05 ‘.’ 0.1 ‘ ’ 1

> anova(metareg.base,metareg.mouse_sex)

df AIC BIC AICc logLik LRT pval QE tau^2 R^2

Full 4 2121.5892 2138.3422 2121.6721 -1056.7946 1544.5557 1.8683

Reduced 2 2141.8600 2150.2366 2141.8848 -1068.9300 24.2709 <.0001 1624.2610 1.9904 6.1356%

**4) Effect of virus genotype as a moderator in the meta-regression model**

> summary(metareg.genotype<-rma(measure="PLO",xi=no_died,ni=no_mice,mods=~genotype-1,data=jemouse,method="DL"))

Mixed-Effects Model (k = 487; tau^2 estimator: DL)

logLik deviance AIC BIC AICc

-1065.7580 1124.7363 2139.5159 2156.2690 2139.5989

tau^2 (estimated amount of residual heterogeneity): 1.9740 (SE = 0.2517)

tau (square root of estimated tau^2 value): 1.4050

I^2 (residual heterogeneity / unaccounted variability): 69.87%

H^2 (unaccounted variability / sampling variability): 3.32

Test for Residual Heterogeneity:

QE(df = 484) = 1606.5513, p-val < .0001

Test of Moderators (coefficients 1:3):

QM(df = 3) = 60.0205, p-val < .0001

Model Results:

estimate se zval pval ci.lb ci.ub

genotype1 0.2320 0.2346 0.9892 0.3226 -0.2277 0.6918

genotype3 0.6280 0.0896 7.0052 <.0001 0.4523 0.8037 ***

genotype5 1.7375 0.5503 3.1573 0.0016 0.6589 2.8161 **

---

Signif. codes: 0 ‘***’ 0.001 ‘**’ 0.01 ‘*’ 0.05 ‘.’ 0.1 ‘ ’ 1

> anova(metareg.base,metareg.genotype)

df AIC BIC AICc logLik LRT pval QE tau^2 R^2

Full 4 2139.5159 2156.2690 2139.5989 -1065.7580 1606.5513 1.9740

Reduced 2 2141.8600 2150.2366 2141.8848 -1068.9300 6.3441 0.0419 1624.2610 1.9904 0.8232%

**5) Effect of virus strain as a moderator in the meta-regression model**

> summary(metareg.virus_strain_resolved<-rma(measure="PLO",xi=no_died,ni=no_mice,mods=~virus_strain_resolved-1,data=jemouse[!is.na(jemouse$virus_strain_resolved),],method="DL"))

Mixed-Effects Model (k = 487; tau^2 estimator: DL)

logLik deviance AIC BIC AICc

-921.9077 837.0358 1921.8154 2085.1577 1928.7952

tau^2 (estimated amount of residual heterogeneity): 1.1281 (SE = 0.1665)

tau (square root of estimated tau^2 value): 1.0621

I^2 (residual heterogeneity / unaccounted variability): 56.46%

H^2 (unaccounted variability / sampling variability): 2.30

Test for Residual Heterogeneity:

QE(df = 449) = 1031.2060, p-val < .0001

Test of Moderators (coefficients 1:38):

QM(df = 38) = 410.2610, p-val < .0001

Model Results:

estimate se zval pval ci.lb ci.ub

virus_strain_resolvedAS6 -1.3689 0.1669 -8.2027 <.0001 -1.6960 -1.0418 ***

virus_strain_resolvedBeijing1 0.8038 0.2172 3.7008 0.0002 0.3781 1.2295 ***

virus_strain_resolvedSA14 2.0914 0.2435 8.5889 <.0001 1.6142 2.5687 ***

virus_strain_resolvedNakayama 0.8327 0.2250 3.7017 0.0002 0.3918 1.2736 ***

virus_strain_resolvedJaTH160 1.6684 0.3541 4.7116 <.0001 0.9744 2.3624 ***

virus_strain_resolvedP20778 -0.2755 0.3293 -0.8365 0.4029 -0.9209 0.3700

virus_strain_resolvedRP9 0.5602 0.2960 1.8923 0.0585 -0.0200 1.1404 .

virus_strain_resolvedP3 1.4631 0.3561 4.1088 <.0001 0.7652 2.1610 ***

virus_strain_resolvedMie/41/2002 -1.1839 0.3336 -3.5485 0.0004 -1.8378 -0.5300 ***

virus_strain_resolvedGP78 2.6788 0.4634 5.7803 <.0001 1.7705 3.5871 ***

virus_strain_resolvedNJ2008 2.1919 0.4262 5.1430 <.0001 1.3566 3.0272 ***

virus_strain_resolvedJaOArS982 -0.0826 0.3883 -0.2127 0.8316 -0.8436 0.6784

virus_strain_resolvedSCYA201201 0.9773 0.3943 2.4785 0.0132 0.2045 1.7501 *

virus_strain_resolved733913 0.8035 0.6230 1.2898 0.1971 -0.4175 2.0246

virus_strain_resolvedCNU/LP2 2.0608 0.5492 3.7523 0.0002 0.9844 3.1372 ***

virus_strain_resolvedM5/ 596 1.5330 0.6259 2.4491 0.0143 0.3062 2.7598 *

virus_strain_resolved78668A 0.4212 0.6950 0.6061 0.5445 -0.9410 1.7835

virus_strain_resolvedYN2016-1 1.0608 0.7702 1.3772 0.1684 -0.4488 2.5704

virus_strain_resolvedMie/40/2004 1.9124 0.5995 3.1898 0.0014 0.7373 3.0875 **

virus_strain_resolvedXZ0934 1.7981 0.6111 2.9424 0.0033 0.6004 2.9958 **

virus_strain_resolvedJEV/SW/GZ/09/2004 -0.1937 0.6822 -0.2840 0.7764 -1.5309 1.1434

virus_strain_resolvedJEV/eq/Tottori/2003 -0.4274 0.4990 -0.8566 0.3917 -1.4054 0.5506

virus_strain_resolvedJaOH0566 -0.9720 0.4953 -1.9625 0.0497 -1.9427 -0.0012 *

virus_strain_resolvedMuar 1.5161 0.7409 2.0463 0.0407 0.0640 2.9682 *

virus_strain_resolvedAT31 2.3116 0.7808 2.9607 0.0031 0.7814 3.8419 **

virus_strain_resolvedJaGAr01 3.6291 1.2613 2.8774 0.0040 1.1571 6.1012 **

virus_strain_resolved7812474 1.9688 0.8355 2.3564 0.0185 0.3312 3.6065 *

virus_strain_resolvedJI 3.4340 1.2635 2.7179 0.0066 0.9577 5.9103 **

virus_strain_resolvedJERG07 3.0445 1.2695 2.3982 0.0165 0.5563 5.5327 *

virus_strain_resolvedJERT07 3.0445 1.2695 2.3982 0.0165 0.5563 5.5327 *

virus_strain_resolvedSarawak 3.0445 1.2695 2.3982 0.0165 0.5563 5.5327 *

virus_strain_resolvedSX09S01 2.5445 1.1495 2.2136 0.0269 0.2916 4.7975 *

virus_strain_resolvedSX06 2.9392 1.2717 2.3112 0.0208 0.4467 5.4317 *

virus_strain_resolvedJaOArB18BP72 0.0665 1.1532 0.0577 0.9540 -2.1937 2.3268

virus_strain_resolved395A/14/SW/IVRI 3.0445 1.7954 1.6958 0.0899 -0.4743 6.5634 .

virus_strain_resolvedJEV/SW/GD/01/2009 -3.0445 1.7954 -1.6958 0.0899 -6.5634 0.4743 .

virus_strain_resolvedSHJEV01 3.0445 1.7954 1.6958 0.0899 -0.4743 6.5634 .

virus_strain_resolvedJaOAr 2.5649 1.8116 1.4158 0.1568 -0.9858 6.1157

---

Signif. codes: 0 ‘***’ 0.001 ‘**’ 0.01 ‘*’ 0.05 ‘.’ 0.1 ‘ ’ 1

> anova(metareg.base,metareg.virus_strain_resolved)

df AIC BIC AICc logLik LRT pval QE tau^2 R^2

Full 39 1921.8154 2085.1577 1928.7952 -921.9077 1031.2060 1.1281

Reduced 2 2141.8600 2150.2366 2141.8848 -1068.9300 294.0447 <.0001 1624.2610 1.9904 43.3219%

**6) Effect of virus dose in PFU as a moderator in the meta-regression model**

> summary(metareg.log10dose_PFU<-rma(measure="PLO",xi=no_died,ni=no_mice,mods=~log10(dose_PFU),data=jemouse[!is.na(jemouse$dose_PFU),],method="DL"))

Mixed-Effects Model (k = 401; tau^2 estimator: DL)

logLik deviance AIC BIC AICc

-860.0603 878.5676 1726.1205 1738.1024 1726.1810

tau^2 (estimated amount of residual heterogeneity): 1.8114 (SE = 0.2404)

tau (square root of estimated tau^2 value): 1.3459

I^2 (residual heterogeneity / unaccounted variability): 65.93%

H^2 (unaccounted variability / sampling variability): 2.93

R^2 (amount of heterogeneity accounted for): 5.73%

Test for Residual Heterogeneity:

QE(df = 399) = 1171.0104, p-val < .0001

Test of Moderators (coefficient 2):

QM(df = 1) = 45.6057, p-val < .0001

Model Results:

estimate se zval pval ci.lb ci.ub

intrcpt -0.4958 0.1716 -2.8892 0.0039 -0.8321 -0.1594 **

log10(dose_PFU) 0.2669 0.0395 6.7532 <.0001 0.1894 0.3443 ***

---

Signif. codes: 0 ‘***’ 0.001 ‘**’ 0.01 ‘*’ 0.05 ‘.’ 0.1 ‘ ’ 1

> summary(metareg.empty.base<-rma(measure="PLO",xi=no_died,ni=no_mice,data=jemouse[!is.na(jemouse$dose_PFU),],method="DL"))

Random-Effects Model (k = 401; tau^2 estimator: DL)

logLik deviance AIC BIC AICc

-881.3693 921.1857 1766.7387 1774.7266 1766.7688

tau^2 (estimated amount of total heterogeneity): 1.9215 (SE = 0.2526)

tau (square root of estimated tau^2 value): 1.3862

I^2 (total heterogeneity / total variability): 67.32%

H^2 (total variability / sampling variability): 3.06

Test for Heterogeneity:

Q(df = 400) = 1224.0726, p-val < .0001

Model Results:

estimate se zval pval ci.lb ci.ub

0.4976 0.0908 5.4780 <.0001 0.3195 0.6756 ***

---

Signif. codes: 0 ‘***’ 0.001 ‘**’ 0.01 ‘*’ 0.05 ‘.’ 0.1 ‘ ’ 1

> anova(metareg.empty.base,metareg.log10dose_PFU)

df AIC BIC AICc logLik LRT pval QE tau^2 R^2

Full 3 1726.1205 1738.1024 1726.1810 -860.0603 1171.0104 1.8114

Reduced 2 1766.7387 1774.7266 1766.7688 -881.3693 42.6182 <.0001 1224.0726 1.9215 5.7299%

**7) Effect of route of administration as a moderator in the meta-regression model**

> summary(metareg.route<-rma(measure="PLO",xi=no_died,ni=no_mice,mods=~route-1,data=jemouse[!is.na(jemouse$route),],method="DL"))

Mixed-Effects Model (k = 487; tau^2 estimator: DL)

logLik deviance AIC BIC AICc

-1045.7721 1084.7647 2109.5443 2147.2387 2109.9216

tau^2 (estimated amount of residual heterogeneity): 1.8823 (SE = 0.2427)

tau (square root of estimated tau^2 value): 1.3720

I^2 (residual heterogeneity / unaccounted variability): 68.65%

H^2 (unaccounted variability / sampling variability): 3.19

Test for Residual Heterogeneity:

QE(df = 479) = 1527.6708, p-val < .0001

Test of Moderators (coefficients 1:8):

QM(df = 8) = 103.9282, p-val < .0001

Model Results:

estimate se zval pval ci.lb ci.ub

routeIC 1.4018 0.1858 7.5424 <.0001 1.0375 1.7660 ***

routeIP+sham IC 1.3075 0.4492 2.9105 0.0036 0.4270 2.1879 **

routeIN 2.0687 0.6722 3.0776 0.0021 0.7513 3.3862 **

routeCONJ 3.0445 1.9944 1.5266 0.1269 -0.8644 6.9534

routeIV 0.3718 0.3256 1.1417 0.2536 -0.2664 1.0099

routeIM 2.0661 0.5294 3.9030 <.0001 1.0286 3.1036 ***

routeIP 0.1891 0.1090 1.7347 0.0828 -0.0246 0.4028 .

routeSC 0.6560 0.2441 2.6871 0.0072 0.1775 1.1344 **

---

Signif. codes: 0 ‘***’ 0.001 ‘**’ 0.01 ‘*’ 0.05 ‘.’ 0.1 ‘ ’ 1

> summary(metareg.empty.base<-rma(measure="PLO",xi=no_died,ni=no_mice,data=jemouse[!is.na(jemouse$route),],method="DL"))

Random-Effects Model (k = 487; tau^2 estimator: DL)

logLik deviance AIC BIC AICc

-1068.9300 1131.0804 2141.8600 2150.2366 2141.8848

tau^2 (estimated amount of total heterogeneity): 1.9904 (SE = 0.2529)

tau (square root of estimated tau^2 value): 1.4108

I^2 (total heterogeneity / total variability): 70.08%

H^2 (total variability / sampling variability): 3.34

Test for Heterogeneity:

Q(df = 486) = 1624.2610, p-val < .0001

Model Results:

estimate se zval pval ci.lb ci.ub

0.6044 0.0830 7.2818 <.0001 0.4417 0.7671 ***

---

Signif. codes: 0 ‘***’ 0.001 ‘**’ 0.01 ‘*’ 0.05 ‘.’ 0.1 ‘ ’ 1

> anova(metareg.empty.base,metareg.route)

df AIC BIC AICc logLik LRT pval QE tau^2 R^2

Full 9 2109.5443 2147.2387 2109.9216 -1045.7721 1527.6708 1.8823

Reduced 2 2141.8600 2150.2366 2141.8848 -1068.9300 46.3157 <.0001 1624.2610 1.9904 5.4306%
