# Supplementary figures and images for: Mouse models of Japanese encephalitis virus infection: A systematic review and meta-analysis using a meta-regression approach
Source: PLoS Negl Trop Dis. 2022 Feb 10;16(2):e0010116. doi: 10.1371/journal.pntd.0010116 (PMC8865681; doi:10.1371/journal.pntd.0010116)

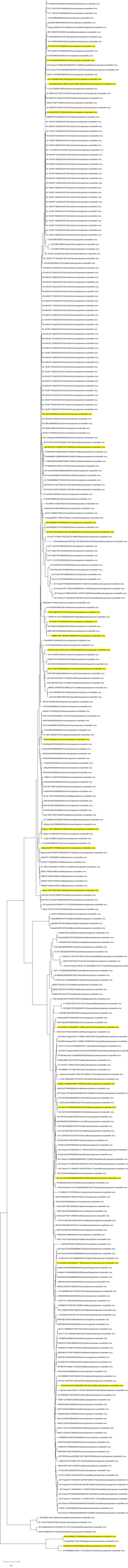

Supplement: S1 Fig — This was created using MEGA-X and genotypes assigned manually. (PDF) [file pntd.0010116.s007.pdf]

**S2 Fig: Locations (countries) of included studies (12 countries, n=127)**

**
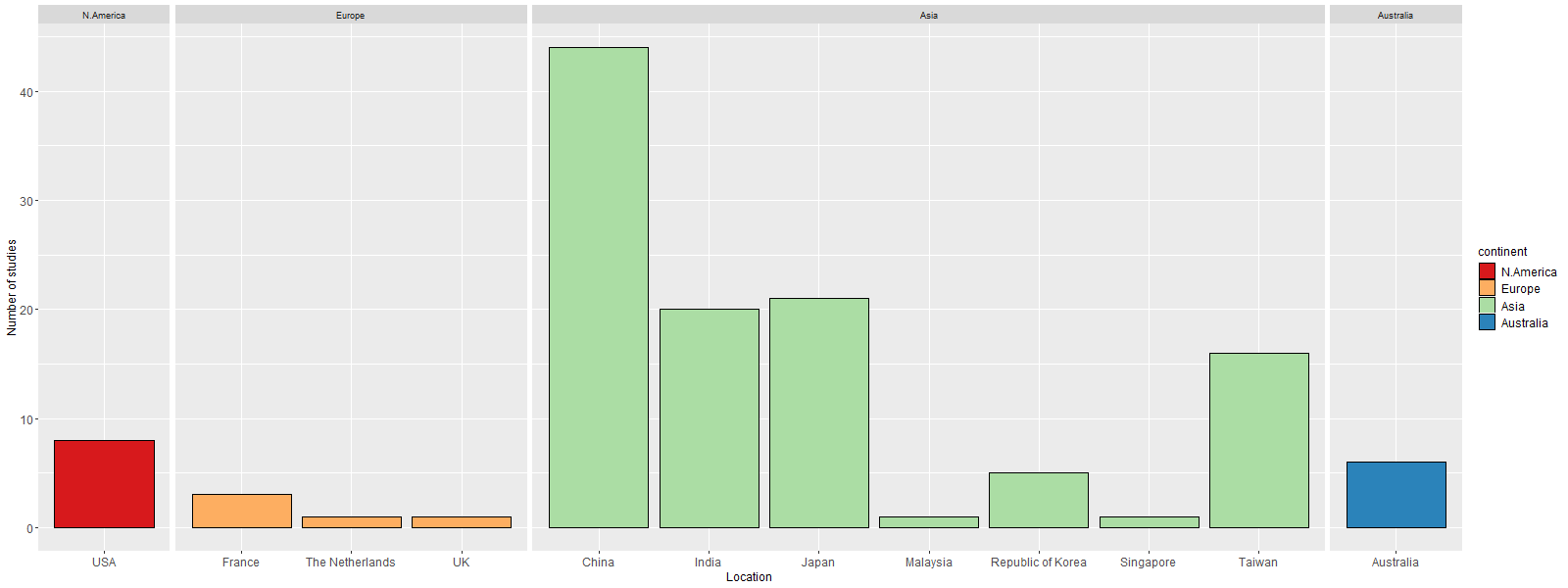
**

Supplement: S2 Fig — (DOCX) [file pntd.0010116.s008.docx]

**S3 Fig: Year of publication of included studies (1970-2020; n=127)**

**
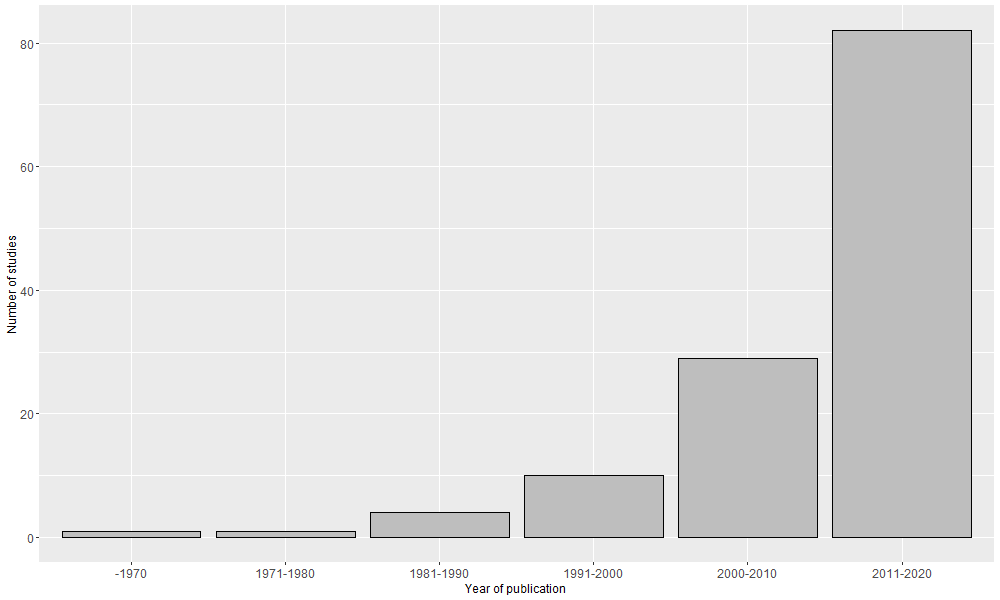
**

Supplement: S3 Fig — (DOCX) [file pntd.0010116.s009.docx]
