# Supplementary material for: Mouse models of Japanese encephalitis virus infection: A systematic review and meta-analysis using a meta-regression approach
Source: PLoS Negl Trop Dis. 2022 Feb 10;16(2):e0010116. doi: 10.1371/journal.pntd.0010116 (PMC8865681; doi:10.1371/journal.pntd.0010116)
Supplement: Attachment — Submitted filename: PNTD-D-21-01120_review.pdf [file pntd.0010116.s010.pdf]

PLOS Neglected Tropical Diseases

Mouse models of Japanese encephalitis virus infection: a systematic review and meta-analysis using a meta-regression approach

--Manuscript Draft--

**Manuscript Number:**	PNTD-D-21-01120
**Full Title:**	Mouse models of Japanese encephalitis virus infection: a systematic review and meta-analysis using a meta-regression approach
**Short Title:**	Mouse models of Japanese encephalitis virus infection
**Article Type:**	Research Article
**Keywords:**	Animal models, systematic review, meta-analysis, metaregression, japanese encephalitis virus, flavivirus, neurological infection.
**Corresponding Author:**	Lance Turtle<br>University of Liverpool<br>Liverpool, UNITED KINGDOM
**Corresponding Author Secondary Information:**	
**Corresponding Author's Institution:**	University of Liverpool
**Corresponding Author's Secondary Institution:**	
**First Author:**	Tehmina Bharucha, BSc MSc MBChB MRCP DTM&H
**First Author Secondary Information:**	
**Order of Authors:**	Tehmina Bharucha, BSc MSc MBChB MRCP DTM&H
	Ben Cleary
	Alice Farmiloe
	Elizabeth Sutton
	Hanifah Hayati
	Peggy Kirkwood
	Layal Al Hamed
	Nadja van Ginneken
	Krishanthi Subramaniam
	Nicole Zitzmann
	Gerry Davies
	Lance Turtle
**Order of Authors Secondary Information:**	
**Abstract:**	Background:<br>Japanese encephalitis (JE) virus (JEV) remains a leading cause of neurological infection across Asia. The high lethality of disease and absence of effective therapies mean that standardised animal models will be crucial in developing therapeutics. However, published mouse models are heterogeneous. We performed a systematic review, meta-analysis and meta-regression of published JEV mouse experiments, to investigate the variation in model parameters, assess homogeneity, and test the relationship of key variables against mortality.<br><br>Methodology/ Principal Findings:<br>A PubMed search was performed up to August 2020. 1991 publications were identified, of which 127 met inclusion criteria, with data for 5026 individual mice across

	487 experimental groups. Quality assessment was performed using a modified CAMRADES criteria, and demonstrated incomplete reporting, with a median quality score of 10/17. The pooled estimate of mortality in mice after JEV challenge was 64.7% (95% confidence interval 60.9 to 68.3) with substantial heterogeneity between experimental groups (I^2 70.1%, df 486). Using meta-regression to identify key moderators, a refined dataset was used to model outcome dependent on five variables: mouse age, virus strain, virus dose (in log 10 PFU) and route of inoculation. The final model reduced the heterogeneity substantially (I^2 37.8%, df 241), explaining 54% of the variability.<br><br>Conclusion/ Significance:<br>This is the first systematic review of mouse models of JEV infection. Better adherence to CAMRADES guidelines may reduce bias and variability of reporting. In particular, sample size calculations were notably absent. We report that mouse strain, age, virus strain, dose and route of inoculation account for much, though not all, of the variation in mortality. This dataset is available for researchers to access and use as a guideline for JEV mouse experiments
**Suggested Reviewers:**	Mario Lobigs<br>The University of Queensland - Saint Lucia Campus: The University of Queensland<br>m.lobigs@uq.edu.au
	Eiji Konishi<br>Mahidol University<br>ekon@biken.osaka-u.ac.jp
	Seong Kug Eo<br>Chonbuk National University: Jeonbuk National University<br>vetvirus@chonbuk.ac.kr
	Emily Sena<br>The University of Edinburgh<br>emily.sena@ed.ac.uk
**Opposed Reviewers:**	
**Additional Information:**	
**Question**	**Response**
**Financial Disclosure**<br><br>Enter a financial disclosure statement that describes the sources of funding for the work included in this submission. Review the submission guidelines for detailed requirements. View published research articles from *PLOS NTDs* for specific examples.<br><br>This statement is required for submission and **will appear in the published article** if the submission is accepted. Please make sure it is accurate.	TB is supported by the University of Oxford and the Medical Research Council [grant number MR/N013468/1]. LT is a Wellcome clinical career development fellow, supported by grant number 205228/Z/16/Z, and the NIHR Health Protection Research Unit in emerging and zoonotic infections (NIHR200907) at University of Liverpool in partnership with Public Health England (PHE), in collaboration with Liverpool School of Tropical Medicine and the University of Oxford. LT is based at University of Liverpool. The views expressed are those of the author(s) and not necessarily those of the NHS, the NIHR, the Department of Health or Public Health England. NZ is supported by the Oxford Glycobiology endowment. The funders had no role in study design, data collection and analysis, decision to publish, or preparation of the manuscript.

**Unfunded studies**<br><br>Enter: *The author(s) received no specific funding for this work.*<br><br>**Funded studies**<br><br>Enter a statement with the following details:<br>• Initials of the authors who received each award<br>• Grant numbers awarded to each author<br>• The full name of each funder<br>• URL of each funder website<br>• Did the sponsors or funders play any role in the study design, data collection and analysis, decision to publish, or preparation of the manuscript?<br>• **NO** - Include this sentence at the end of your statement: *The funders had no role in study design, data collection and analysis, decision to publish, or preparation of the manuscript.*<br>• **YES** - Specify the role(s) played.<br><br>* typeset	
**Competing Interests**<br><br>Use the instructions below to enter a competing interest statement for this submission. On behalf of all authors, disclose any competing interests that could be perceived to bias this work—acknowledging all financial support and any other relevant financial or non-financial competing interests.<br><br>This statement **will appear in the published article** if the submission is accepted. Please make sure it is accurate. View published research articles from *PLOS NTDs* for specific examples.	The authors have declared that no competing interests exist.

**NO authors have competing interests**<br><br>Enter: *The authors have declared that no competing interests exist.*<br><br>**Authors with competing interests**<br><br>Enter competing interest details beginning with this statement:<br><br>*I have read the journal's policy and the authors of this manuscript have the following competing interests: [insert competing interests here]*<br><br>* typeset<br><br>This statement is required for submission and **will appear in the published article** if the submission is accepted. Please make sure it is accurate and that any funding sources listed in your Funding Information later in the submission form are also declared in your Financial Disclosure statement.	
**Data Availability**<br><br>Authors are required to make all data underlying the findings described fully available, without restriction, and from the time of publication. PLOS allows rare exceptions to address legal and ethical concerns. See the PLOS Data Policy and FAQ for detailed information.	Yes - all data are fully available without restriction

A Data Availability Statement describing where the data can be found is required at submission. Your answers to this question constitute the Data Availability Statement and **will be published in the article**, if accepted.<br><br>**Important:** Stating 'data available on request from the author' is not sufficient. If your data are only available upon request, select 'No' for the first question and explain your exceptional situation in the text box.<br><br>Do the authors confirm that all data underlying the findings described in their manuscript are fully available without restriction?	
**Describe where the data may be found in full sentences. If you are copying our sample text, replace any instances of XXX with the appropriate details.**<br><br>• If the data are **held or will be held in a public repository**, include URLs, accession numbers or DOIs. If this information will only be available after acceptance, indicate this by ticking the box below. For example: *All XXX files are available from the XXX database (accession number(s) XXX, XXX.).*<br>• If the data are all contained **within the manuscript and/or Supporting Information files**, enter the following: *All relevant data are within the manuscript and its Supporting Information files.*<br>• If neither of these applies but you are able to provide **details of access elsewhere**, with or without limitations, please do so. For example:<br><br>*Data cannot be shared publicly because*	Data are available as supplemental information

of [XXX]. Data are available from the XXX Institutional Data Access / Ethics Committee (contact via XXX) for researchers who meet the criteria for access to confidential data.<br><br>*The data underlying the results presented in the study are available from (include the name of the third party and contact information or URL).*<br>• This text is appropriate if the data are owned by a third party and authors do not have permission to share the data.<br><br>* typeset	
Additional data availability information:	

To the Editor,

Plos NTD

July 2021

To the Editors,

Re: **Mouse models of Japanese encephalitis virus infection: a systematic review and meta-analysis using a meta-regression approach**

We would be grateful if you could consider our submission describing a systematic review and meta-analysis of mice models of Japanese encephalitis virus.

Japanese encephalitis (JE) is a devastating disease, with no promising antiviral candidate therapies. Treatment trials that have been conducted were only powered to detect very large treatment effects. There are still around 100,000 cases per year despite the availability of vaccines. In order to have any hope of developing new treatments, standardised animal models will be needed. However, mouse models of JE are variable in their characteristics, with many contradictory findings in the literature. For this reason, we performed a systematic review, meta-analysis and meta-regression of published JEV mouse experiments, to investigate the variation in model parameters, assess homogeneity, and test the relationship of key variables against mortality.

This is the first report, we are aware of, of a systematic review and meta-analysis of mouse models of JE. We demonstrate an abundance of experimental work in this field. However, reporting was frequently incomplete and there was considerable variability in outcomes. We provide recommendations for researchers to improve standardisation of study design and reporting of studies. Furthermore, all the data have been provided in supporting files.

Please let us know if you need further information.

Kind regards,

The Authors

Corresponding author - Dr Lance Turtle. Email: lance.turtle@liverpool.ac.uk. Address: Institute of Infection, Veterinary and Ecological Sciences, Faculty of Health and Life Sciences, University of Liverpool, Liverpool, UK.

Manuscript

Click here to access/download;Manuscript;260721_Manuscript_PLOS

Full title: Mouse models of Japanese encephalitis virus infection: a systematic review and meta-analysis using a meta-regression approach

Short title: Mouse models of Japanese encephalitis virus infection

Authors and Affiliations:

Tehmina Bharucha[1,2+]

Ben Cleary[3+]

Alice Farmiloe[3]

Elizabeth Sutton[3]

Hanifah Hayati[6]

Peggy Kirkwood[3]

Layal Al Hamed[3]

Nadja van Ginneken[4]

Krishanthi Subramaniam[3]

Nicole Zitzmann[1]

Gerry Davies[3,5]

Lance Turtle*[3,5]

1. Oxford Glycobiology Institute, Department of Biochemistry, University of Oxford, Oxford, UK.
2. Lao-Oxford-Mahosot Hospital-Wellcome Trust Research Unit (LOMWRU), Microbiology Laboratory, Mahosot Hospital, Vientiane, Lao PDR
3. Institute of Infection, Veterinary and Ecological Sciences, Faculty of Health and Life Sciences, University of Liverpool, Liverpool, UK.
4. Department of Primary Care and Mental Health, University of Liverpool, Liverpool, UK.
5. Tropical and Infectious Disease Unit, Liverpool University Hospitals NHS Foundation Trust (member of Liverpool Health Partners), Liverpool, UK.
6. Department of Neurology, Faculty of Medicine, Gadjah Mada University, Dr Sardjito Hospital, Yogyakarta, Indonesia

[+]Joint first author

*Corresponding author lance.turtle@liverpool.ac.uk

Author emails

t.bharucha@doctors.org.uk

bencleary94@gmail.com

alice.farmiloe@hotmail.co.uk
esutton@liverpool.ac.uk
hanifahfh@gmail.com
peggykirkwood@hotmail.com
L.alhamed@hotmail.com
nadjavg@liverpool.ac.uk ORCID ID: 0000-0002-4843-2617
K.Subramaniam@liverpool.ac.uk ORCID ID: 0000-0002-1734-9351
nicole.zitzmann@bioch.ox.ac.uk
gerrydavies@doctors.org.uk
lance.turtle@liverpool.ac.uk

Abstract

Background:

Japanese encephalitis (JE) virus (JEV) remains a leading cause of neurological infection across Asia. The high lethality of disease and absence of effective therapies mean that standardised animal models will be crucial in developing therapeutics. However, published mouse models are heterogeneous. We performed a systematic review, meta-analysis and meta-regression of published JEV mouse experiments to investigate the variation in model parameters, assess homogeneity and test the relationship of key variables against mortality.

Methodology/ Principal Findings:

A PubMed search was performed up to August 2020. 1991 publications were identified, of which 127 met inclusion criteria, with data for 5026 individual mice across 487 experimental groups. Quality assessment was performed using a modified CAMARADES criteria and demonstrated incomplete reporting with a median quality score of 10/17. The pooled estimate of mortality in mice after JEV challenge was 64.7% (95% confidence interval 60.9 to 68.3) with substantial heterogeneity between experimental groups (I^2 70.1%, df 486). Using meta-regression to identify key moderators, a refined dataset was used to model outcome dependent on five variables: mouse age, mouse strain, virus strain, virus dose (in log_{10}PFU) and route of inoculation. The final model reduced the heterogeneity substantially (I^2 37.8%, df 241), explaining 54% of the variability.

Conclusion/ Significance:

This is the first systematic review of mouse models of JEV infection. Better adherence to CAMARADES guidelines may reduce bias and variability of reporting. In particular, sample size calculations were notably absent. We report that mouse age, mouse strain, virus strain, virus dose and route of inoculation account for much, though not all, of the variation in mortality. This dataset is available for researchers to access and use as a guideline for JEV mouse experiments.

Author Summary

Japanese encephalitis (JE) virus (JEV) remains a leading cause of brain infection across Asia, resulting in considerable death and disability. No effective treatment exists. Mouse models are fundamental to evaluate novel treatments. We aimed to perform the first systematic literature review and data synthesis of JEV infection in mouse models.

We identified an abundance of experimental data in the field, with 127 studies meeting the inclusion criteria involving a total of 5026 individual mice. Overall, 65% of mice died after JEV infection. However there was incomplete reporting in publications and considerable variability in the results.

In summary, the findings support the ongoing use of mouse models of JEV infection and inform researchers in the field in refining their experiments. Key factors affecting variation in mortality across studies that need to be carefully considered in study design are mouse age, mouse strain, virus strain, virus dose and route of inoculation. We highlight the need for researchers to adhere to reporting guidelines in preparing manuscripts for publication.

Introduction

Japanese encephalitis (JE) virus (JEV) remains a leading cause of neurological infection across Asia (1, 2). The single stranded, positive sense RNA virus is a member of the genus *Flavivirus*, and groups into five genotypes (I-V) based on genomic sequence, with human infections primarily caused by genotypes I and III (3). JEV transmission occurs in enzootic cycles between mosquitoes, pigs and birds, with human infection arising primarily from infected *Culex* spp. mosquitoes (4). There are an estimated 100,000 cases of JEV infection per annum (5), with recent dynamic modelling suggesting that in 2021 there will be 23,600 deaths and a loss of 2,500,000 disability adjusted life years (5, 6). Moreover, JE predominantly affects children in endemic areas and has devastating socioeconomic consequences.

A handful of randomised clinical trials of treatments for JE have been performed to date, however no effective treatment has been identified (7). Multiple vaccines exist and are recommended by the World Health Organisation (WHO) (8). Although recent efforts have strengthened JE vaccination programs, still only 12 of 24 endemic countries include JE vaccine in routine immunisation policies; even then, it is not uniformly nationwide, with vaccine coverage in targeted areas reported to be as low as 39% (1). As viremia in JE is too low to propagate, humans are considered dead-end hosts and the infection is zoonotic, which provides additional challenges as vaccination alone is therefore not sufficient for JE eradication (4).

The high lethality of JE and absence of effective therapies mean that animal models are crucial in developing our understanding of the disease and therapeutic prospects. There have been reports published of experiments using JEV in mouse models dating back to 1935 when the virus was first isolated (9, 10). Mice are frequently the preferred model for studying human infections due to their low-cost, timely reproduction and variability (11). In the last decade, many studies of JEV have used animal models to address a wide variety of different questions, such as the role of various components of the immune system in protection from JEV, pathogenesis of JE and for testing treatments. These studies have contributed greatly towards further understanding JE pathogenesis (7). However, mouse models of JE have not been standardised and can be highly variable across laboratories contributing to contradictory results (12).

No systematic review of mouse models of JE has been conducted. Therefore, in order to understand which model parameters account for variation, and to ensure homogeneous reporting of results, we conducted a systematic review of published experiments using JEV in mouse models. We hypothesised that virus strain, virus dose, route of administration, mouse strain, mouse age and mouse sex would influence lethality. We therefore aimed to test the relationship of these variables on mortality from JEV infection in mice and to develop guidance on the set up and reporting of mouse models of JE.

Methods

The study adhered to PRISMA guidelines for systematic reviews and the protocol is included in supplementary data (S1_Data). A PubMed search was performed using the terms ("encephalitis, japanese"[MeSH Terms] OR ("encephalitis"[All Fields] AND "japanese"[All Fields]) OR "japanese encephalitis"[All Fields] OR ("japanese"[All Fields] AND "encephalitis"[All Fields]) OR "je"[All Fields] OR "jev"[All Fields]) AND ("mice"[MeSH Terms] OR "mice"[All Fields] OR "mouse"[All Fields] OR "mice"[MeSH Terms] OR "mice"[All Fields] OR “mus”[All Fields]). The search date ranged from 1935 (the year of first isolation of JEV) to August 2020 and only English language text was included. Retrieved references were downloaded into EndNote for the removal of duplicate studies and the abstracts and/or full-text screened by two authors independently for eligibility as per the criteria detailed below, see Figure 1 for the PRISMA flow diagram.

The inclusion criteria were any publication that included an experiment meeting the following criteria: 1. JEV was inoculated into mice; 2. virus dose was reported; 3. JEV strain or source was reported; 4. immunocompetent mice were used and the strain was reported; 5. mortality was reported as either death or humane endpoint (primary analysis & outcome measures, secondary outcome measures 1 and 2) or other pathological outcome reported (secondary outcome 3); 6. published in English; and 7. primary research. Additional data were also extracted (see S1_Data) but did not serve as an exclusion from the primary analysis. Studies were excluded if they reported inoculation using non-pathogenic JEV (for example the vaccine strain SA14-14-2) only, or if data on individual animals was not reported and it was not possible to extract the

data. In order to minimise non-specific immune effects, data were collected only on groups of mice that received JEV only, and no other material. For example, data were frequently derived from reports that tested a treatment or vaccine, in these cases only the control group was extracted.

Quality assessment and data extraction were performed by two authors independently, using standardised excel sheet proformas. The quality of studies was assessed based on ten standard quality measures used previously for animal model meta-analyses by CAMARADES (the Collaborative Approach to Meta-Analysis and Review of Animal Data in Experimental Studies) (12). Additional data extracted as study quality measures were: statement of the intent of the experiments conducted, mouse strain used, virus used, dose of virus given, route of inoculation given, age of mice at inoculation explicitly stated or easily calculated (e.g., "mice were immunised at 6 weeks of age … and challenged 2 weeks after immunisation") and cell type/tissue that the virus was derived from. Each study was allocated a score out of 17 based on these quality measures.

Data were analysed using R version 4.0.2 (13). Variables of interest were plotted against mortality at the level of individual experiments. Publication bias was explored using funnel plots. Individual and aggregated forest plots were used to summarise data, stratified by key variables. Meta-regression was used to quantify the impact of experiment-level covariates on heterogeneity of outcomes. The generalised R^2 (explained variance) and I^2 (total heterogeneity/variability) statistics, likelihood ratio test, and Akaike's Information Criterion (AIC) were used to judge model fit. Routine regression diagnostics were used to test model assumptions. A multivariable analysis was performed using all of the variables in a single model to estimate mortality. Sensitivity analyses were used to consider the

strength of effect of each variable individually using a forward stepwise approach, i.e. assessing the iterative effect of including them in the model.

Results

Summary

The initial search identified 1991 articles, of which 127 were included in the review, detailed in the PRISMA flow diagram (Figure 1). Data were available on 5026 individual mice in 487 experimental groups challenged with JEV. Studies involved a median of two (IQR 1-4; range 1-84) groups where JEV was inoculated into mice with no other treatment or substance; one study performed by Miura *et al.* in 1988 (14) represented an outlier with 84 groups involving a total of 527 mice. Experimental groups included a median of 10 (IQR 5-12; range 2-112) mice; 18 (3.7%) with over 20 mice, and two (0.4%) with over 50 mice. No study performed in the last 5 years included an experimental group with over 20 mice. Between 1970 and 2020 studies were conducted in 12 countries, primarily in Asia (China, India, Japan, Republic of Korea, Singapore, Malaysia, Taiwan), but also in Australia, Europe (U.K., France, The Netherlands) and N. America (U.S.A.) (S6_Figure and S7_Figure). Eighty-four last authors were identified in 54 institutions, with almost half (25; 46%) of institutions publishing multiple studies.

Figure 1: PRISMA flow diagram of study selection

Compared with CAMARADES criteria, several pieces of information in specific areas were not reported. Less than 50% of studies included statements that allocation to experimental group was random, treatment and outcome were blinded to investigator,

neuroprotective anaesthesia was used and no conflict of interests were present. No study reported having performed a sample size calculation or that the temperature was controlled during experiments. Median quality score was 10 (IQR 9-11, range 7-13) across the 17 criteria (figure 2). Data extracted are displayed in Table 1, with details of the variables and missing study-level characteristics.

Figure 2: Quality assessment of included studies

Table 1: Variables with data extracted

Variable	Details	No. (%) of studies with missing data, i.e. no reporting
Year of publication	6 categories	0 (0%)
Last author	84 categories	0 (0%)
Institution	54 categories	0 (0%)
Country	12 categories	0 (0%)
Mouse strain*	14 categories	0 (0%)
Mouse age	4 categories	2 (1.6%)
Mouse sex	2 categories	59 (56%)
Virus genotype	3 categories	0 (0%)
Virus strain*	38 categories	0 (0%)
Virus dose in PFU*	log10PFU continuous	29 (22.8%)
Route of administration	8 categories	0 (0%)

*The variable was a criteria for inclusion in the systematic review. For the purpose of meta-regression analysis, doses in tissue culture infective dose (TCID50) were converted to plaque forming units (PFU) and doses in lethal dose (LD) were excluded.

The global estimate of mortality in mice after JEV challenge, i.e. the base meta-regression model, was 64.7% (95% confidence interval 60.9 to 68.3). There was substantial

heterogeneity (I^2 70.1%, df 486) between experimental groups. Therefore, we next determined the influence of key variables to develop a final meta-regression model.

Analysis of the moderating effect and interactions of key variables

Mouse strain: Fourteen different mouse strains were used in the studies, see Figure 3A. Six strains were used for the vast majority (4771; 95%) of studies; C57BL/6, BALB/c, Swiss, C3H/He, ICR and ddY mice. There were less than 100 mice (range 6-65) studied for each of the eight remaining strains. The mortality of JEV challenge in different mouse strains was highly variable (Figure 3B). Overall, BALB/c, Swiss and ICR were more susceptible to JEV than other mouse strains and ddY was more resistant. Incorporating mouse strain as a moderator in the base meta-regression model reduced the variability of the base model (I^2 63.6%, S2_Data). Analysis of the top six mouse strains (S3_Data) suggested interactions between the variables, for example mortality in ICR mice is higher than C57BL/6; however the median age of the mice used in experiments with the different strains is different at 14 (IQR 3-21) vs 42 (IQR 35-95) days respectively.

Figure 3A: Summary of the number of mice of different mouse strains used; **Figure 3B:** Mortality from JEV challenge in different strains of mice used

Mouse age: Mouse age was categorised into months. Mice used in JEV challenge studies were predominantly immature (3754/5025; 75%), i.e. less than 3 months old (Figure 3C). Mortality declined with the age of the mice (Figure 3D). Incorporating mouse age as a moderator in the base meta-regression model reduced the variability of the base model (I^2 65.2%, S2_Data).

Figure 3C: Summary of the ages of mice used; **Figure 3D** Mortality from JEV challenge in mice of different ages used

Mouse sex: Sixty-eight (54%) of studies reported the mouse sex, data were missing for 2686 mice (53%). The vast majority of mice for which data were available were female (2299; 98%). Mortality in male vs. female mice following JEV challenge was not significantly different (Figure 3E). Incorporating mouse sex as a moderator in the base meta-regression model minimally reduced the variability of the base model (I^2 68.7%, S2_Data).

Figure 3E: Mortality from JEV challenge in male and female mice

Virus genotype: Three of the five known JEV genotypes were used in studies; predominantly genotype 3 (4407; 88%), to a lesser extent genotype 1 (515; 10%) and rarely genotype 5 (104; 2%). Mortality of mice challenged with different genotypes is

shown in Figure 3F. Incorporating virus genotype as a moderator in the base meta-regression model minimally reduced the variability of the base model (I^2 69.9%), see S2_Data.

Figure 3F Mortality in mice challenged with different JEV genotypes

Virus strain: Thirty-eight different JEV strains were used in the studies (Figure 4A); 7 (18%) genotype 1, 29 (77%) genotype 3 and 2 (5%) genotype 5. Twenty of 127 studies (16%) reported accession numbers but none reported sequencing data to confirm the virus used. There were 16 strains with identifiable accession numbers, 12 full and 4 partial sequences; there did not appear to be any particular genetic selection bias given the relationship to other virus isolates on a phylogenetic tree (S4_Figure).

Figure 4A: Summary of the number of mice that received different JEV strains

Ten JEV strains were used in less than 100 mice each, such that 13 JEV strains represented 4004 (80%) of the mice included in the review. The mortality of JEV challenge with different virus strains was highly variable (Figure 4B). Incorporating virus strain as a moderator in the base meta-regression model reduced the variability of the base model (I^2 56.5%, S2_Data). Analysis of the top 13 JEV strains (S5_Data) suggested interaction between the variables, for example mice challenged with AS6 have significantly lower mortality than those challenged with Beijing1, however the median dose of AS6 is over 6000 times lower than that of Beijing1.

Figure 4B: Mortality in mice challenged with different JEV strains

Virus dose: Ninety-three (73%) studies reported the dose in plaque forming units (PFU), 28 (22%) in lethal dose (LD), 5 (4%) in median tissue culture infectious dose (TCID50) and 1 (1%) in median cell culture infectious dose (CCID50). TCID50 was converted to PFU (PFU = 0.7 x TCID50) and the analysis was performed with the virus dose in log10PFU, using this approach there were missing data for 1239 (25%) mice included in the review. There was a normal distribution of doses used in the studies (Figure 5a). There was no clear relationship between dose and mortality, however performing a subgroup analysis by route did demonstrate an association, see Figures 5B and 6. Incorporating virus dose in PFU as a moderator in the base meta-regression model reduced the variability of the base model (I^2 65.9%), see S2_Data.

Figure 5A: Summary of the number of mice that received different doses; **Figure 5B:** Mortality in mice following JEV challenge against dose in $\log_{10}$PFU

Figure 6: Mortality in mice following JEV challenge against dose in $\log_{10}$PFU grouped by the route of inoculation.

Route of administration: Eight routes of JEV challenge were used across the included studies; intracerebral (IC), sub-cutaneous (SC), intranasal (IN), conjunctival (CONJ), intravenous (IV), intramuscular (IM), intraperitoneal (IP) and IP with sham IC, see Figure 7A. IN and CONJ were used in only 64 (1.3%) and 10 (0.2%) mice respectively. There

was no significant difference in mortality in mice challenged by different routes of administration, see Figure 7B. Incorporating virus dose in PFU as a moderator in the base meta-regression model minimally reduced the variability of the base model (I^2 68.7%), see S2_Data. Analysis of the top 6 routes of administration, see S8_Data, suggests interaction between these variables.

Figure 7A: Summary of the number of mice that were challenged by different routes of administration; **Figure 7B:** Mortality in mice challenged by different routes of administration

Interactions

Analysis was performed to examine interactions of key variables. There were no clearly identifiable interactions. This was in part due to a lack of data for all the categories; for example, as seen in Figure 8, investigators use mice of the same age for specific mouse strains.

Figure 8: Boxplot of the distribution of ages of mice across mice strains used in the included studies

Meta-regression analysis

In order to reduce instability in the model due to missing study-level characteristics and to better explore interactions between the variables, a sub-group analysis was performed

using the top six mouse strains, top 13 virus strains and top six routes of inoculation. The reduced dataset included 242 (49.7%) of the experimental groups and 2562 (51.0%) of the mice in the full dataset. The pooled estimate of mortality in mice after JEV challenge, i.e. the base meta-regression model for the reduced dataset was 64.3 (95% confidence interval 58.9 to 69.4, Figure 9). There was substantial heterogeneity (I^2 70.0%, df 241) between experimental groups. In view of the analysis results of the key variables and biological plausibility of their role in moderating the effect, the final model using the reduced dataset included the top six mouse strains, mouse age in months, 13 most frequently used virus strains, virus dose in PFU and the six commonest routes of administration. The final model reduced the heterogeneity substantially (I^2 37.8%, df 241). This confirmed our starting hypothesis that mouse strain, mouse age, route of administration, virus dose and virus strain account for much of the variation in mortality in mouse models of JE.

Figure 9: Forest plot of the studies included in the final meta-regression model

Discussion

The results highlight the wealth of data available on mouse models of JE, with 127 studies meeting the eligibility criteria for inclusion, totalling 5026 mice in 487 experimental groups. The pooled estimate for mortality was 64.7% (95% confidence interval 60.9 to 68.3%). Mouse strain, mouse age, route of administration, virus dose and virus strain account for much of the variation in mortality in mouse models of JE. The large number of mice studied to date, the significant unresolved heterogeneity across studies and the ongoing knowledge gaps in our understanding of JE (particularly the lack of effective treatment) underlines the importance of this systematic review to inform future research. In particular, the analysis aims to refine future mouse models of JEV infection, to improve the quality of future studies and reduce unnecessary replication. The extracted data has been made publicly available to enable researchers to perform their own analysis.

Quality assessment was relatively low with a median quality score of 10 (IQR 9-11, range 7-13) across the 17 criteria. However, there was an improvement in the score over time. This highlights the need to reduce risk of bias through detailed reporting, following CAMARADES guidelines. In particular, there is a need for performance and reporting of blinding, randomisation and *a priori* sample size calculations. None of the studies were excluded based on these quality assessments. Beyond the quality assessment criteria, analysis of data extracted demonstrated missing data for study-level characteristics, most strikingly for reporting of mouse sex in 59 (56%) studies. Furthermore, accession numbers were only reported in 20 (16%) studies with no study reporting sequencing of

the actual JEV strain used, which is important given the potential for sequence variability after serial passage in culture.

The number of mice per experimental group has become more consistent with time, as researchers are more aware of the need to reduce unnecessary waste. Nonetheless, it is always a balance between sufficient power versus minimising numbers used/ sacrificed. In mouse experiments, it is typical to use 5 mice for inbred strains and 8-10 for the outbred, however based on our findings, a power calculation suggests that larger numbers are needed. For example, to detect a halving of mortality from an intervention in experiments of this kind with an overall mortality of 64.7%, 35 mice are needed per group to give 80% power at the 5% significance level.

Analysis of moderating effect of key variables

Mouse strain: Unsurprisingly, C57BL/6 (black inbred strain; 1637 - 33%) and BALB/c (white inbred strain; 1409 – 28%) were the most common strains used; these are well-characterised, reproducible, easily available and cheap (15). C57BL/6 mice have a tendency to generate Th-1 immune responses, whereas BALB/c are skewed towards Th-2 responses. Furthermore, the data were consistent with existing dogma that BALB/c are more susceptible to infection than C57BL/6, with median survival (IQR) 0% (0-30) and 50% (17-80) respectively. Although the analysis was restricted to six strains with more than 100 mice in each group (C57BL/6, BALB/c, Swiss, C3H/He, ICR and ddY mice; 4771- 95%), all were susceptible to JEV infection. It was not possible to resolve individual mouse

strains, as there is significant variation even between sub-strains based on breeding history, original parent strains, and source locations (18).

Mouse age: JEV predominantly affects children in endemic areas, which most likely reflects early life exposure leading to immunity by adulthood (16), though adults are also susceptible to JE upon first exposure (17). The blood brain barrier matures with age; to what extent this is relevant to human disease is not clear, but in mice this is clearly relevant, with some investigators using a sham IC injection in order to disrupt the blood brain barrier and allow encephalitis to develop (18). The systematic review provides support for this, as mortality reduced with age.

Virus strain: Variation of the virus is a plausible explanation for variation between models. Genotype 3 (G3) JEV remains the most commonly isolated JEV genotype from human cases; accepting that virus isolation from cases is a rare event and is also the most frequently used in mouse models of JEV infection (19). Many different strains of JEV have also been used in mouse models. These are rarely sequenced by the investigators, meaning it was not possible to compare sequences and/or resolve the strains used. The top 13 virus strains, relatively well-characterised sequences, constituted 80% of all mice included in the systematic review, and the viral strain accounted for 14% of the variability of the models.

Virus dose: Although the ideal reporting of virus dose is in quantified infectious units, or PFU, many papers do not use this measure and instead report the TCID50 or LD50. Genome copies measured by RT-qPCR may also serve as a measure of viral inoculum, though in practice this is rarely used. TCID50 is a direct measure of infectivity and can be used to approximate PFU whereas LD50 is confounded

by being influenced by the outcome variable in our analysis and cannot be approximated in this way. For this reason there were significant missing data (1239 - 25% mice). Nonetheless, in view of the biological plausibility of the moderating effect of dose, the dose in log_{10}PFU was included in the final model.

Route of inoculation: The preferred route of inoculation involves the one with the best external validity, that which is technically straightforward to perform safely with a hazard group 3 virus such as JEV and provides a robust and reproducible infection. The IP method was most widely used (2811 – 56% mice), followed by IC (924 -18% mice) and then SC (539 – 11%). The meta-regression analysis showed a minimal impact of the route of inoculation on reducing heterogeneity, however further exploration demonstrated that this was due to interaction of other variables. It was notable that there was an association between the proximity of the route of inoculation to the brain and mortality (p value < 0.001).

A refined dataset of 2562 mice was produced and a final meta-regression model run using mouse age, mouse strain, virus strain, virus dose in log_{10}PFU and route of inoculation. The final model reduced the heterogeneity substantially (I^2 37.8%, df 241) such that 54% of the variability was explained. Despite this analysis, nearly half of the variability in mouse models of JE remained unexplained, leaving significant room for variation due to individual laboratories, and therefore also room for improvement in standardisation of these important and useful models.

Undoubtedly there are inherent limitations in mouse models of infectious diseases that affect the external validity of the findings. Furthermore, the missing data for combinations

of the different key variables reduces the internal validity. Nonetheless, this review still represents the comprehensive body of data on mouse models of JE assembled to date. We summarise our final recommendations in Table 2. Attention to performance and reporting of experiments on key factors identified in this review will reduce heterogeneity and enable standardisation of models. This is critical to enable evaluation of novel therapeutics.

Table 2: Final recommendations

- Power calculations are crucial to ensure that experiments are appropriately designed to detect effects of interventions
- Factors that affect variability in outcomes and need careful attention in study design include mouse strain, mouse age, virus strain, virus dose and route.
- Virus strains used in experiments need to be sequenced and the data included in publications.
- The data has been made publicly available and serves to inform future experiments in this field.

Acknowledgments

We thank Alan Barrett (The University of Texas Medical Branch) for useful discussion.

References

1. Heffelfinger JD, Li X, Batmunkh N, Grabovac V, Diorditsa S, Liyanage JB, et al. Japanese Encephalitis Surveillance and Immunization - Asia and Western Pacific Regions, 2016. MMWR Morb Mortal Wkly Rep. 2017;66(22):579-83.
2. Bharucha T, Shearer FM, Vongsouvath M, Mayxay M, de Lamballerie X, Newton PN, et al. A need to raise the bar - A systematic review of temporal trends in diagnostics for Japanese encephalitis virus infection, and perspectives for future research. Int J Infect Dis. 2020.
3. Knipe DM, Howley P. Fields Virology. Philadelphia, UNITED STATES: Wolters Kluwer Health; 2013.
4. Pearce JC, Learoyd TP, Langendorf BJ, Logan JG. Japanese encephalitis: the vectors, ecology and potential for expansion. J Travel Med. 2018;25(suppl_1):S16-S26.
5. Quan TM, Thao TTN, Duy NM, Nhat TM, Clapham H. Estimates of the global burden of Japanese encephalitis and the impact of vaccination from 2000-2015. eLife. 2020;9:e51027.
6. Li X, Mukandavire C, Cucunubá ZM, Echeverria Londono S, Abbas K, Clapham HE, et al. Estimating the health impact of vaccination against ten pathogens in 98 low-income and middle-income countries from 2000 to 2030: a modelling study. The Lancet. 2021;397(10272):398-408.
7. Turtle L, Solomon T. Japanese encephalitis — the prospects for new treatments. Nature Reviews Neurology. 2018;14:298.
8. Who. Japanese Encephalitis Vaccines: WHO position paper, February 2015-Recommendations. Vaccine. 2016;34(3):302-3.
9. Webster LT. JAPANESE B ENCEPHALITIS VIRUS: ITS DIFFERENTIATION FROM ST. LOUIS ENCEPHALITIS VIRUS AND RELATIONSHIP TO LOUPING ILL VIRUS. J Exp Med. 1938;67(4):609-18.
10. Hayashi M. Übertragung des Virus von Encephalitis epidemica japonica auf Affen*. Psychiatry and Clinical Neurosciences. 1933;1(1):419-65.
11. Buer J, Balling R. Mice, microbes and models of infection. Nature Reviews Genetics. 2003;4(3):195-205.
12. Ritskes-Hoitinga M, Leenaars M, Avey M, Rovers M, Scholten R. Systematic reviews of preclinical animal studies can make significant contributions to health care and more transparent translational medicine. Cochrane Database of Systematic Reviews. 2014(3).
13. Team RC. R: A language and environment for statistical computing. In: Computing RFfS, , editors. Vienna, Austria.2020.
14. Miura K, Goto N, Suzuki H, Fujisaki Y. Strain difference of mouse in susceptibility to Japanese encephalitis virus infection. Jikken dobutsu Experimental animals. 1988;37(4):365-73.
15. Sarkar S, Heise MT. Mouse Models as Resources for Studying Infectious Diseases. Clinical therapeutics. 2019;41(10):1912-22.
16. Campbell G, Hills S, Fischer M, Jacobson J, Hoke C, Hombach J, et al. Estimated global incidence of Japanese encephalitis:. Bulletin of the World Health Organization. 2011;89:766-74.
17. Halstead SB, Grosz CR. Subclinical Japanese encephalitis. I. Infection of Americans with limited residence in Korea. American journal of hygiene. 1962;75:190-201.
18. Hase T, Dubois DR, Summers PL. Comparative study of mouse brains infected with Japanese encephalitis virus by intracerebral or intraperitoneal inoculation. International journal of experimental pathology. 1990;71(6):857-69.
19. Schuh AJ, Ward MJ, Leigh Brown AJ, Barrett AD. Dynamics of the emergence and establishment of a newly dominant genotype of Japanese encephalitis virus throughout Asia. J Virol. 2014;88(8):4522-32.

Supporting information captions

S1_Data: Protocol: A systematic review of mouse models of Japanese encephalitis

S2_Data: R code for meta-regression analysis

S3_Data: Analysis of top 6 mouse strains

S4_Figure: JEV phylogenetic tree including all complete genome sequences uploaded to GenBank and partial sequences if used in studies with sequences used in studies highlighted

S5_Data: Analysis of top 13 virus strain

S6_Figure: Locations (countries) of included studies (12 countries, n=127)

S7_Figure: Year of publication of included studies (1970-2020; n=127)

S8_Data: Analysis of top 6 routes

Figure

Click here to access/download;Figure;Figure 1.png

PRISMA 2009 Flow Diagram

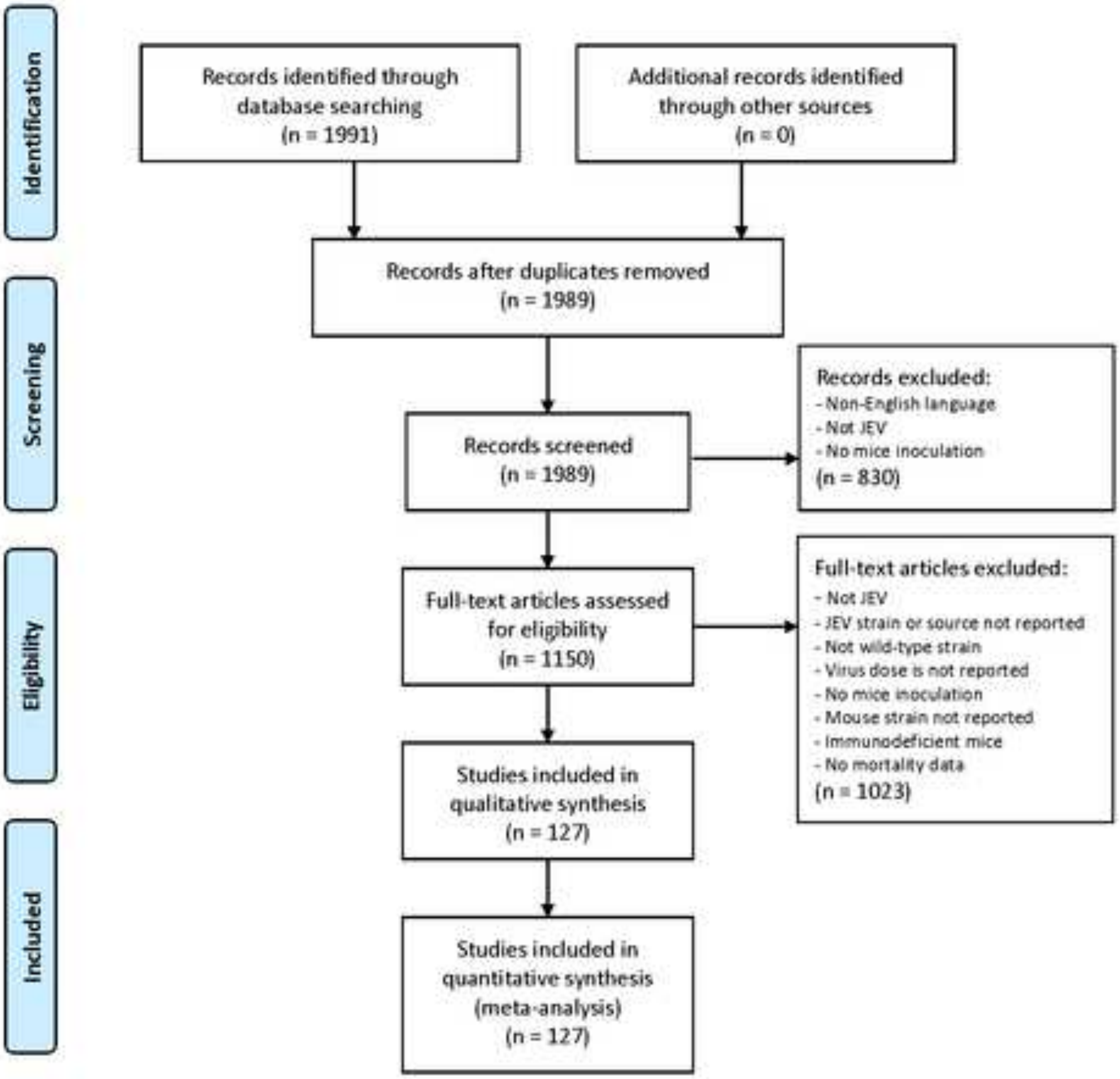

From: Moher D, Liberati A, Tetzlaff J, Altman DG, The PRISMA Group (2009). *Preferred Reporting Items for Systematic Reviews and Meta-Analyses*: The PRISMA Statement. PLoS Med 6(7): e1000097. doi:10.1371/journal.pmed1000097

For more information, visit www.prisma-statement.org.

Figure

Click here to access/download;Figure;Figure 2.png

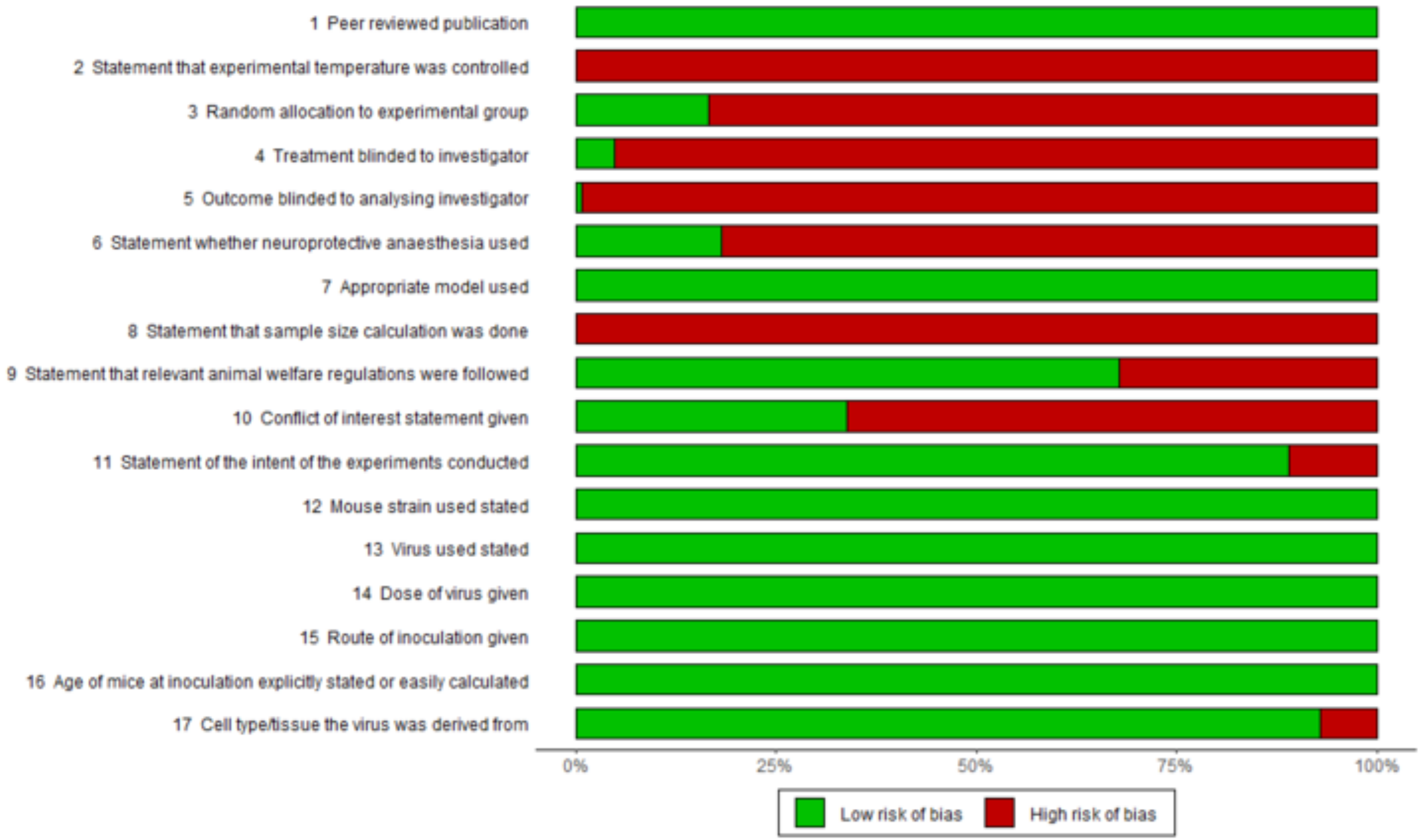

Figure

Click here to access/download;Figure;Figure 3.png

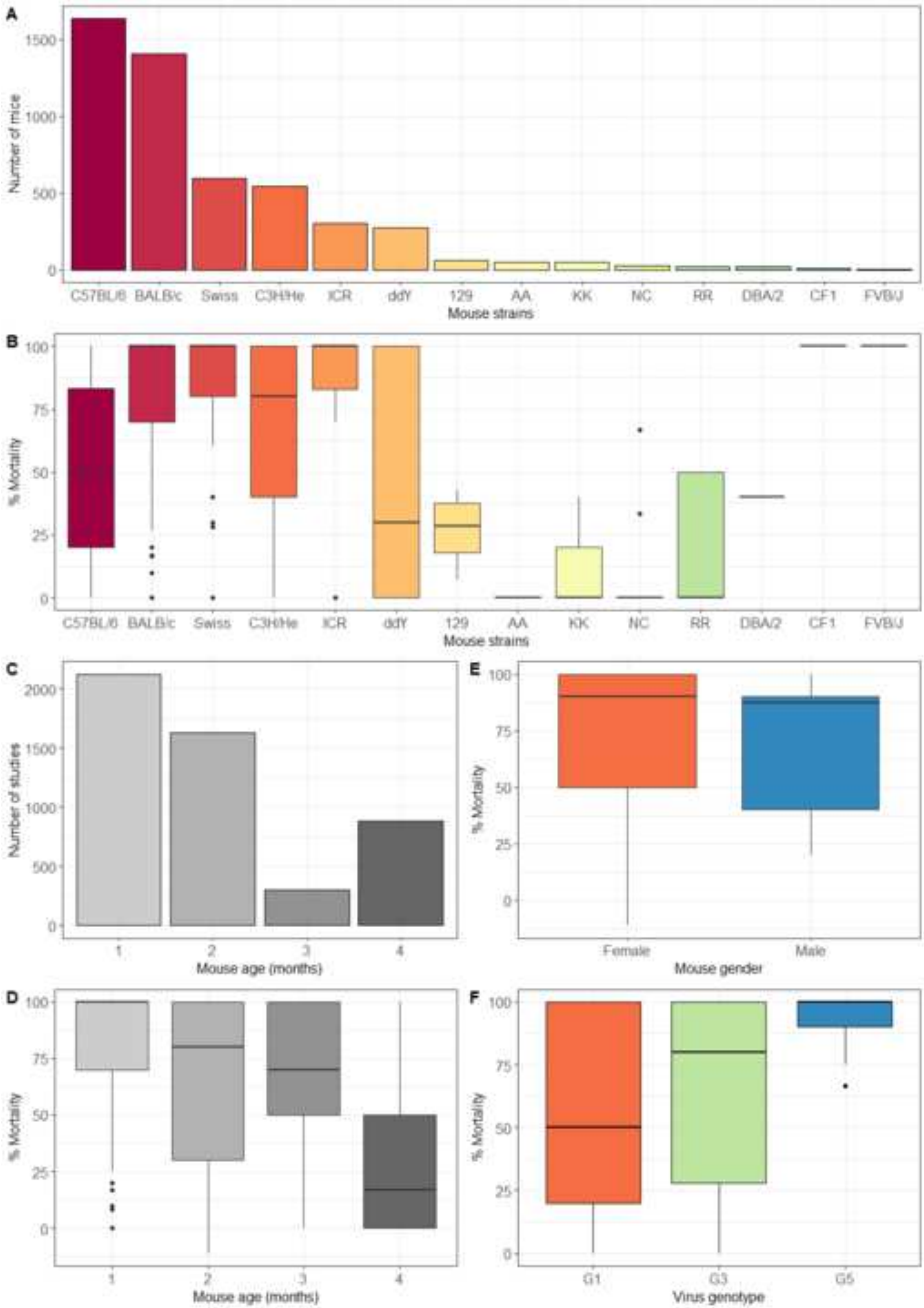

Figure Click here to access/download;Figure;Figure 4.png

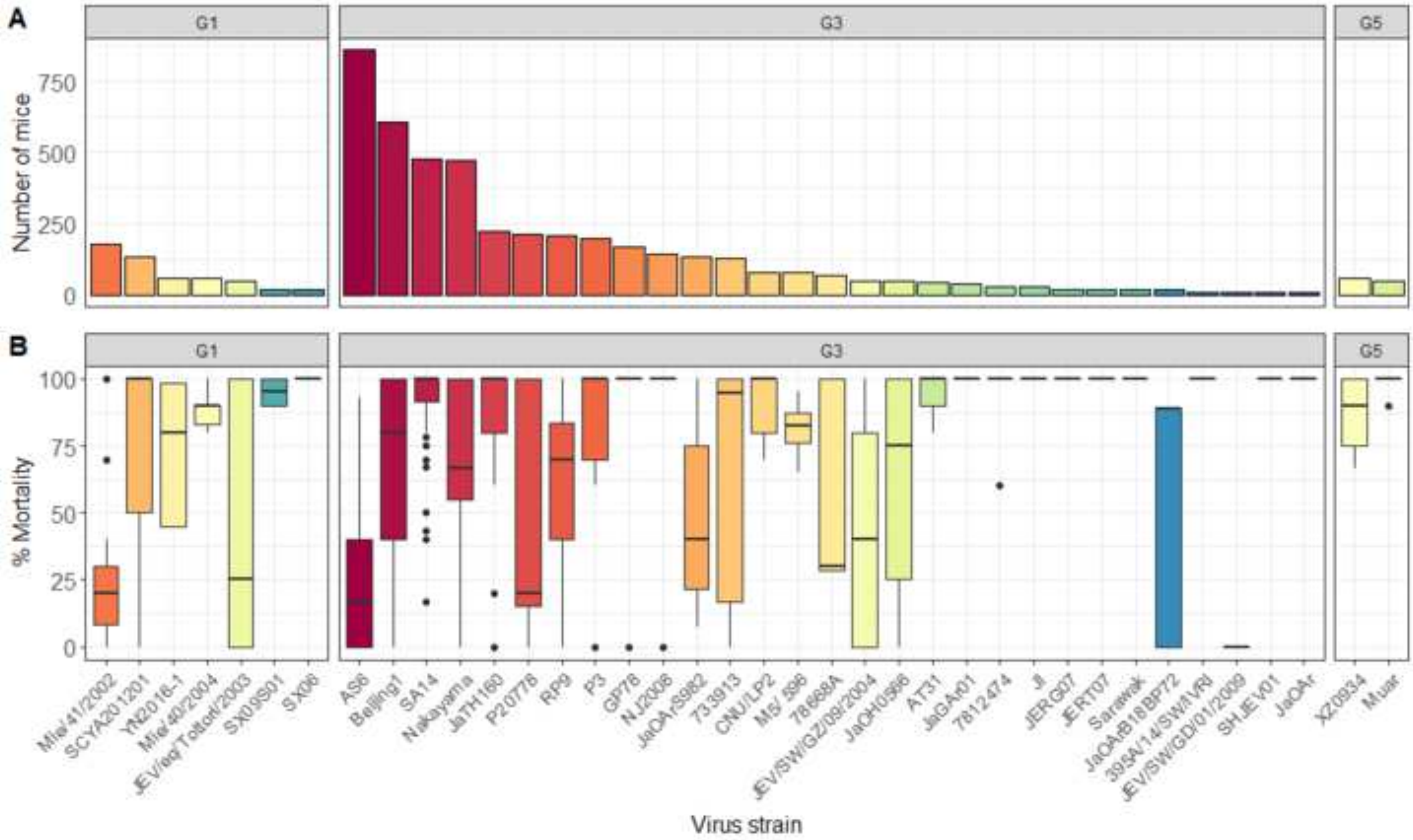

Figure

Click here to access/download;Figure;Figure 5.png

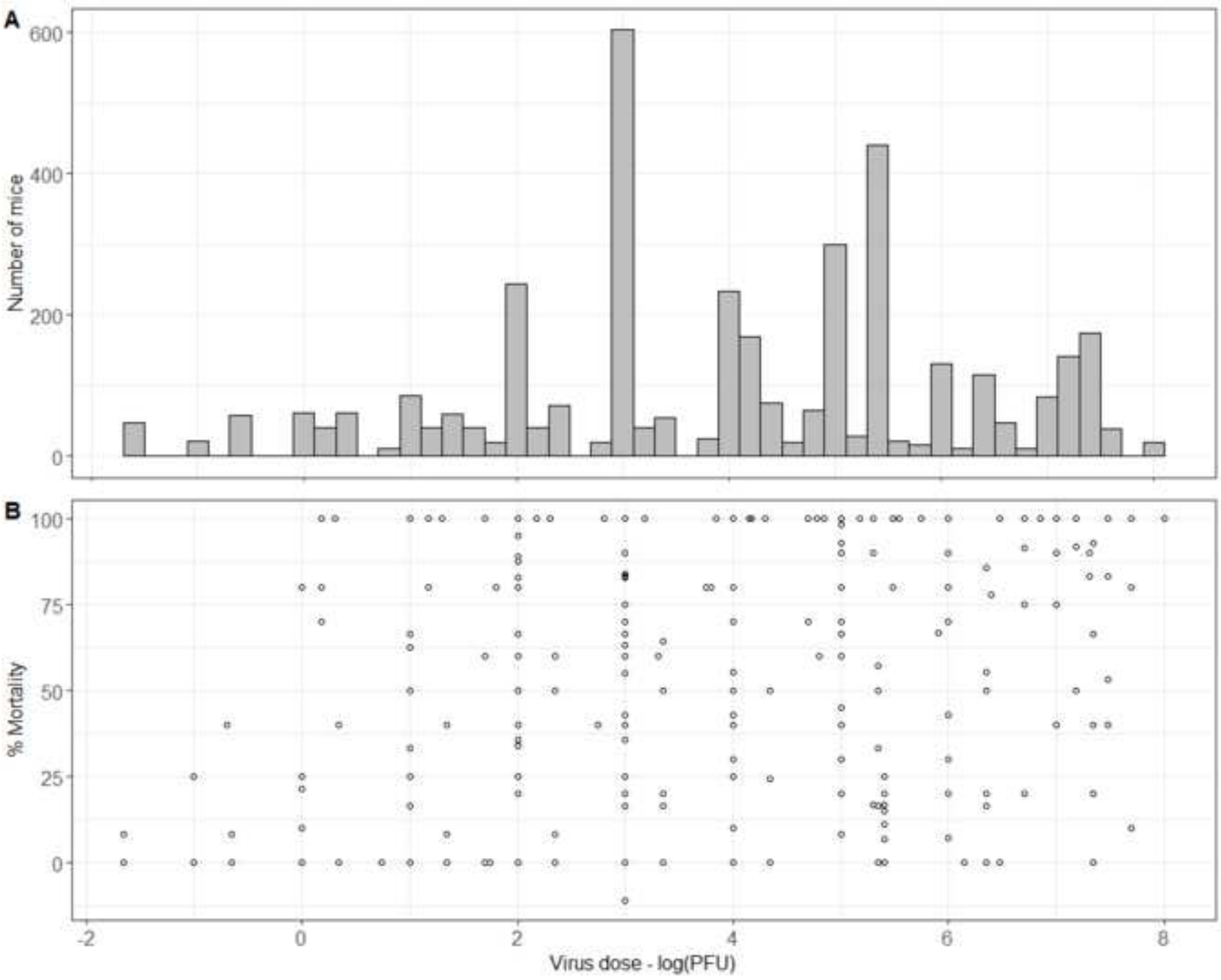

Figure

Click here to access/download;Figure;Figure 6.png

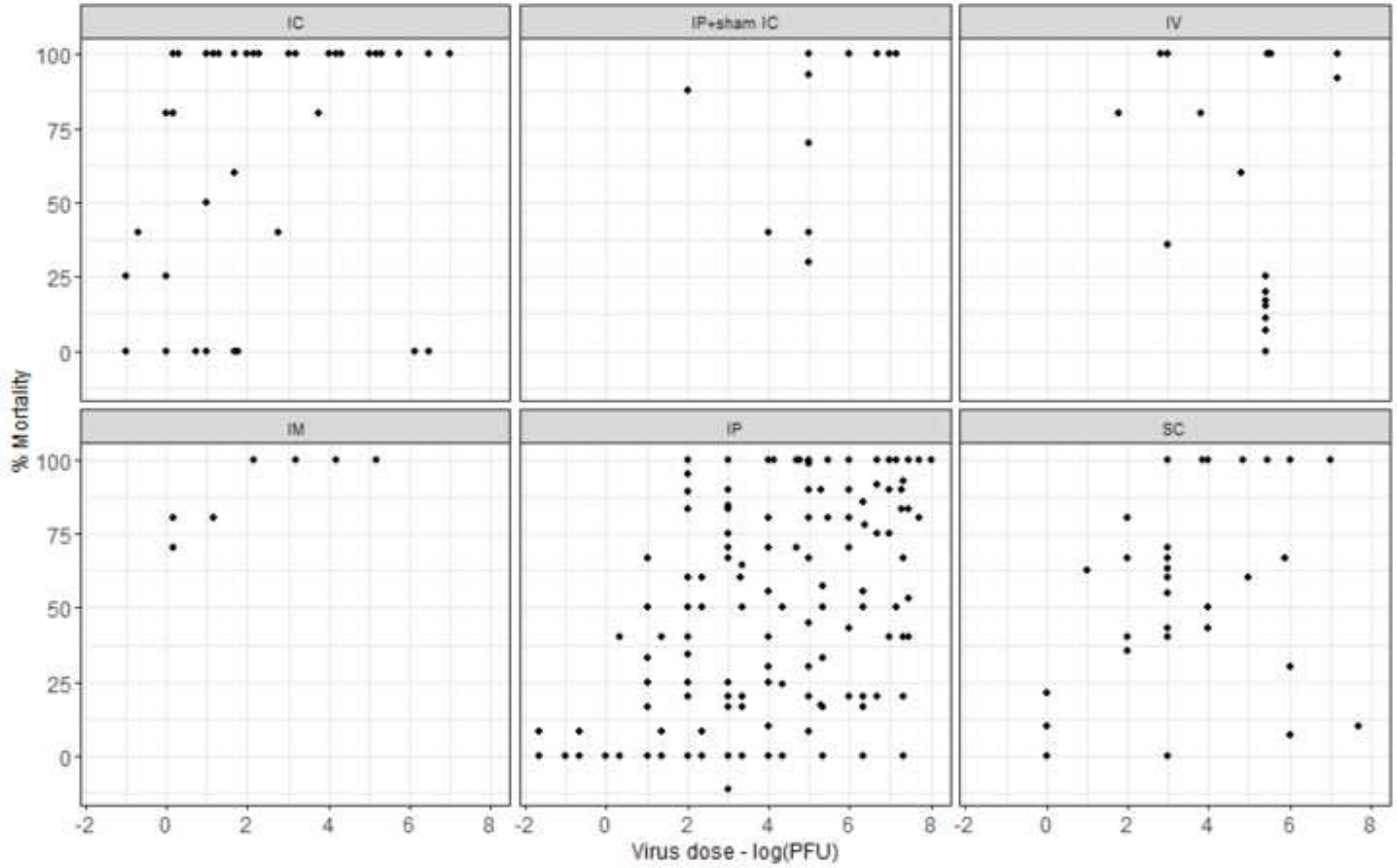

Figure

Click here to access/download;Figure;Figure 7.png

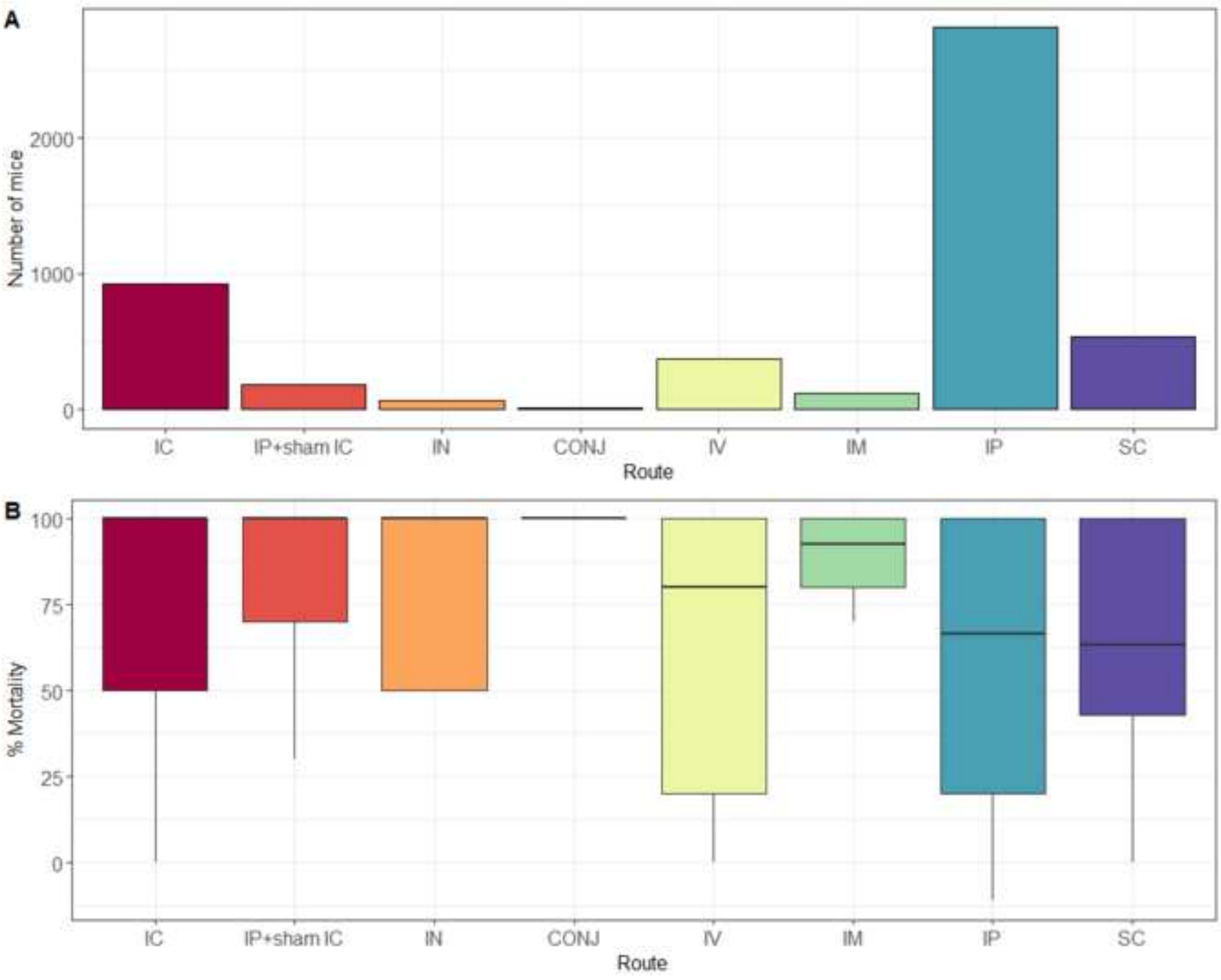

Figure

Click here to access/download;Figure;Figure 8.png

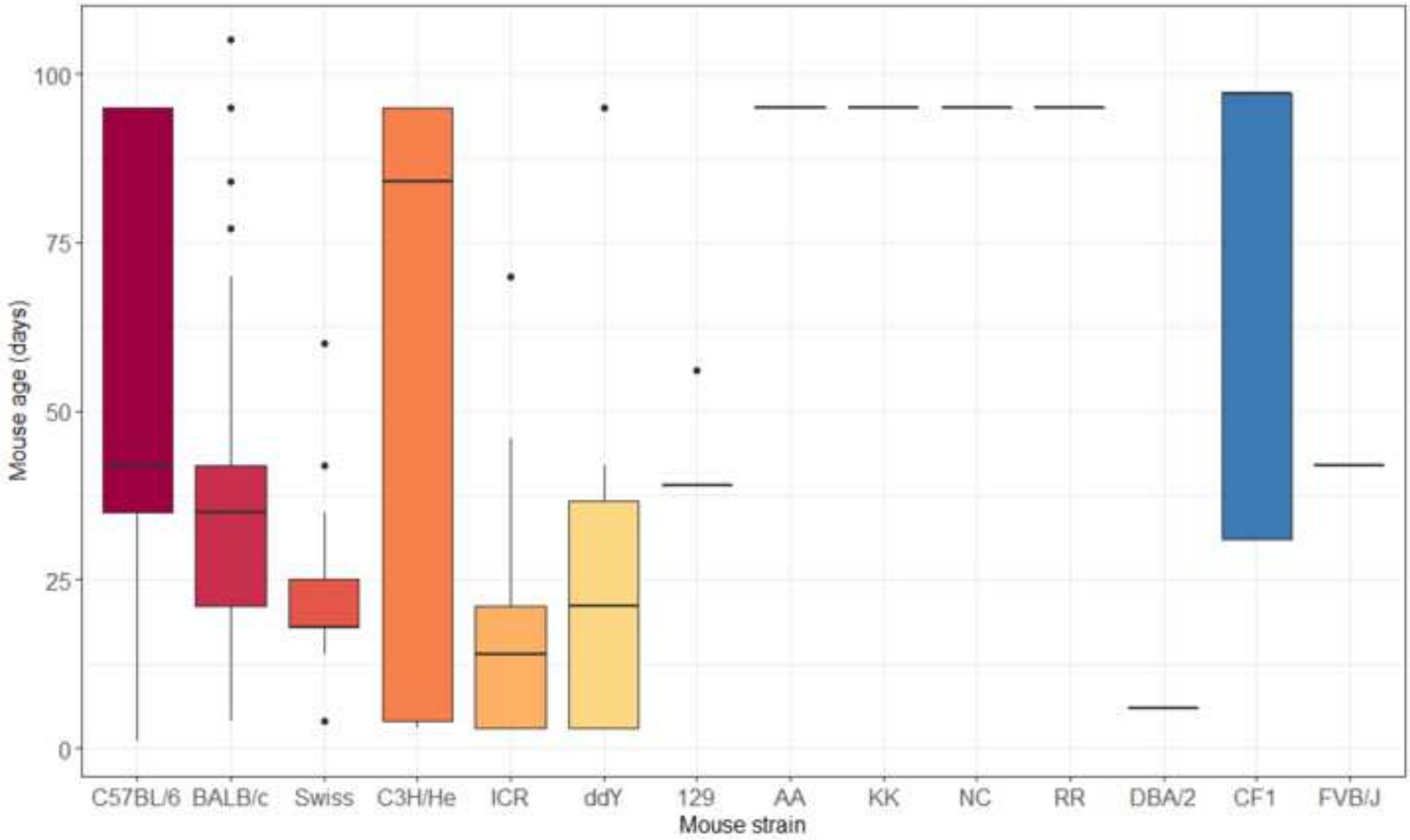

Figure

Click here to access/download;Figure;Figure 9.pdf

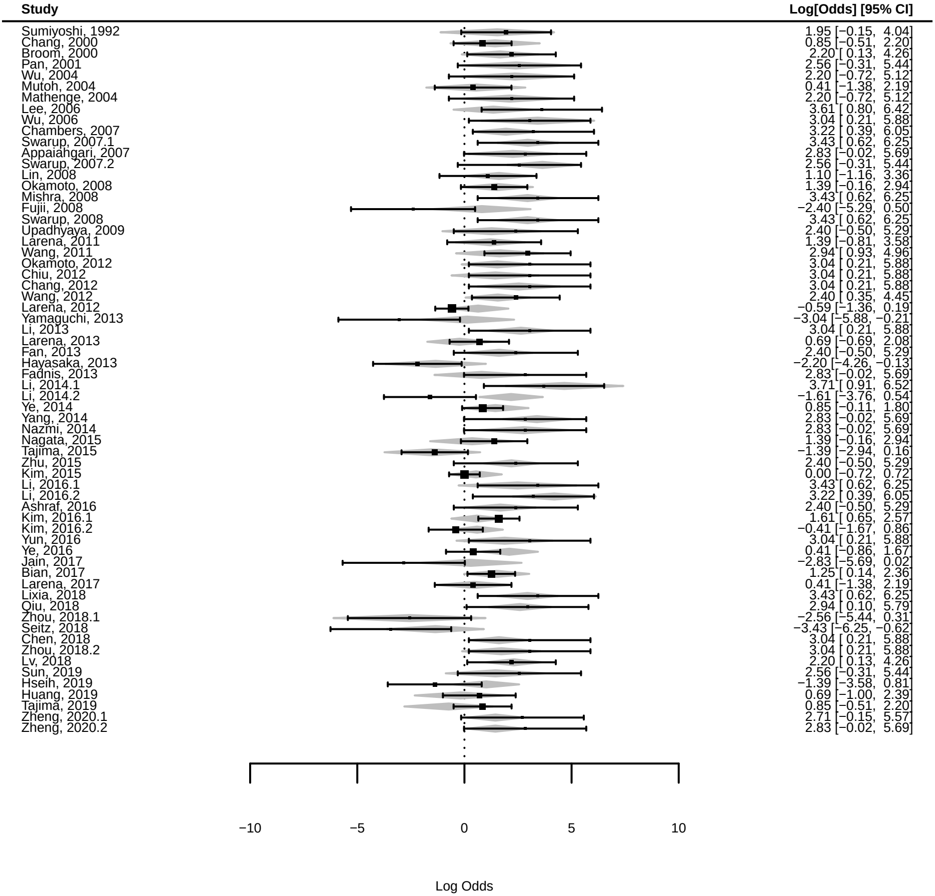

Click here to access/download
Supporting Information
S1_Data.docx

Click here to access/download
Supporting Information
S2_Data.docx

Click here to access/download
Supporting Information
S3_Data.xlsx

Click here to access/download
Supporting Information
S4_Figure.pdf

Click here to access/download
Supporting Information
S5_Data.xlsx

Click here to access/download
Supporting Information
S6_Figure.docx

Click here to access/download
Supporting Information
S7_Figure.docx

Click here to access/download
Supporting Information
S8_Data.xlsx

Click here to access/download
Supporting Information
S9_Data.xlsx
