# Supplementary material for: Mouse models of Japanese encephalitis virus infection: A systematic review and meta-analysis using a meta-regression approach
Source: PLoS Negl Trop Dis. 2022 Feb 10;16(2):e0010116. doi: 10.1371/journal.pntd.0010116 (PMC8865681; doi:10.1371/journal.pntd.0010116)
Supplement: Attachment — Submitted filename: 221121_Response to reviewers.docx [file pntd.0010116.s011.docx]

To the Editor,

Plos NTD

November 2021

### To the Editors,

Re: Response to reviewers’ comments - PNTD-D-21-01120

Article title: Mouse models of Japanese encephalitis virus infection: a systematic review and meta-analysis using a meta-regression approach

We are grateful to the reviewers for their detailed evaluation and useful comments on the manuscript. We have made suggested amendments. Please see the point-by-point responses in blue text below. We would be pleased to offer further information, if required.

Kind regards,

The Authors

Corresponding author - Dr Lance Turtle. Email: [lance.turtle@liverpool.ac.uk](mailto:lance.turtle@liverpool.ac.uk). Address: Institute of Infection, Veterinary and Ecological Sciences, Faculty of Health and Life Sciences, University of Liverpool, Liverpool, UK.

Reviewer's Responses to Questions

Reviewer #1: The plan for data collection, criteria for data inclusion/exclusion, parameters to be analysed from every published work are clearly stated. The methodological details have been extensively described and raw data provided in supplementary material.

Reviewer #2: The objective of the study has been clearly stated and is well substantiated.

The hypothesis is outlined clearly and the study design is appropriate. Detailed methods in the form of a protocol are presented  in Supplementary Data 1.The population is clearly defined, where there is a potential issue with the sample size, these have been discussed.
I am not particularly qualified to comment on the statistical analysis, however to my best knowledge the authors have used standard methodologies for such studies.

As this is a meta analysis, I have no particular concerns regarding the ethical and regulatory requirements. The included works are cited as appropriate and the entire data set has been made available.

--------------------

Reviewer #1: There is stepwise description of results, aiming at decreasing heterogeneity in observations and hence arriving at most common factors which are likely to be important for consideration of mice as experimental animals for JE infection. This is done systematically. However there are following issues which could be noted and revised:

1. In general, there are titles for the figures and no legends. In some cases there is not enough description of results in the text leaving figures incomprehensible. This may be amended.

Author’s response:

- We appreciate the suggestions; figure legends have been added to improve the clarity of individual figure.

2. More specifically, in Figure 9, forest plot, what are the rhomboids in gray colour depicting?

Author’s response:

- The rhomboids/diamonds in Figure 9 were depicting the fitted value for each study as per the default output from the R package metafor (<https://rdrr.io/cran/metafor/man/forest.rma.html>). These have been removed to simplify the figure.

3. Figure 2 details the parameters used for scoring papers into adhering to norms and standards. However, there are low risk and high risk bias categories shown in each parameter. The reason for defining low vs. high risk is not clear. Is simple absence of documentation in papers, such as 'formal sample size calculation', a criterion for division?

Author’s response:

The reviewer is correct to point out that the application of a checklist like this can seem draconian, and is not necessarily indicative of a poor quality study. However, it is also true that we cannot properly assess the quality if these measures are not reported. We draw attention to the fact that these metrics in the JE animal model literature are poorly reported, in the hope that we can stimulate authors in the field to report these better. As per the CAMARADES group, fulfilling criteria equates to ‘low risk of bias’ and not fulfilling the criteria equates to ‘high risk of bias’. A statement to this effect has been added to the methods section and the Figure 2 legend: “For each quality measure, fulfilling the criteria was interpreted as a score of 1 and ‘low risk of bias’.”

4. What is meant by 'experimental temperature was controlled'? [Fig 2] Does it mean mouse housing temperature? Does it mean mice were treated with anti-pyretics?

Author’s response:

Sorry that this was not more clearly articulated. The statement that ‘experimental temperature was controlled’ refers to the housing temperature of the mice, as per the CAMARADES checklist. Figure 2 has been amended; experimental temperature is now referred to as housing temperature.

5. Can fitted lines/curves be shown for Fig 5b and Fig 6 to improve visual appreciation?

Author’s response:

A fitted line has been added to Figure 5b. We are concerned that adding a fitted line to Figure 6 may be misleading due to the sparsity of data and have not added one, however if you are keen for us to do so then please let us know.

6. Some of the parameters mentioned in Table 2, are more appropriate for human work, and less for mouse work; e.g. treatment and outcome blinded to analysing investigator. They do not seem to be relevant in the final analysis. Can justification for their inclusion in the first place be provided?

Author’s response:

As stated in the experimental design assistant on the National Centre for Replacement, Refinement and Reduction of Animals in Research (NC3Rs) guidance (<https://eda.nc3rs.org.uk/experimental-design-blinding>): “Blinding is a research strategy that involves keeping the people who conduct the experiment, assess the outcome and collect the data unaware of the treatment allocation. It is used as a means to limit biased interpretation of treatment.” Although this may have more of an effect in human studies, it is nevertheless a fundamental tenet of high quality experimental work, both in animal models and in human studies performing laboratory work for trial endpoints. Those carrying out the experimental work should be blinded to the treatment allocation and clinical outcome.

7. The authors also document that no paper included in this metanalysis undertook formal sample size calculation. While in principle this is correct, the conflict between statistical requirements for robust data and IACUC imposed restrictions are one major reason for this deficiency observed. This, if acceptable to the authors as a reason, may be elaborated in the text.

Author’s response:

The reviewer is correct to point out the potential tension here between stringent study design and the use of potentially larger numbers of animals. However, performing fewer experiments, but making those better designed and powered, would be an acceptable alternative, and indeed would even be an improvement. We have edited the text in the discussion to reflect the comment as follows: “The number of mice per experimental group has become more consistent with time, as researchers are more aware and institutional guidance mandates reductions in experimental group sizes.”

Reviewer #2: The analysis presented in consistent with the analysis plan. The results appear to be completely presented, however, they are not clear.
Figure legends do not sufficiently describe the figures

Author’s response:

As per reviewer 1, we have expanded the figure legends to improve the clarity of individual figures.
--------------------

Reviewer #1: Refinement in analysis has been achieved by eliminating parameters or by eliminating outliers etc. and it makes sense for providing relatively robust guidelines for future use of mice as a model system for JE infection. The final figure, Figure 9, provides a more reliable analysis for future use where mouse strain, mouse age, route of administration, virus dose and virus strain are identified as parameters on which the outcome depends.

Reviewer #2: The conclusions are well supported by the data presented in the study. The limitations of the analysis are discussed, but alternative explanations or expansion of the hypothesis to encompass other factors - such as end point of the study has not been discussed in sufficient detail.

Author’s response:

We are grateful to the reviewer for this insightful comment. In fact, when we initially conceived this study, this had been an intention. However, as we have shown, the quality of reporting is highly heterogeneous, and it was not possible to consistently assess any parameters other than death. Mouse death we considered the most objective and consistently reported, and was therefore selected as the endpoint for this study. Unfortunately, there was insufficient reported data to study alternative end points such as mouse behaviour or neurological presentations.

The authors make very clear recommendations for future studies - both in the experimental plan as well as reporting of results. This is important and is likely to impact the experimental approach to Japanese Encephalitis Virus pathogenesis  in mouse models. The authors have positioned this study within the public health context and have clearly presented the relevance of the study.

Author’s response:

We thank the reviewer for their kind comments.

--------------------

Reviewer #1: Minor explanations and modifications are needed which are listed above under Results section.

Reviewer #2: Perhaps it would be good to present S2 data as a table within the main manuscript?

Author’s response:

- We have added a table (Table 2) in the manuscript to show the role of variables included in the final model.

Figure 1: PRISMA workflow, in the screening stage the numbers do not add up

Author’s response:

We apologise for this error- The PRISMA flow diagram has been updated; there were 839 studies excluded at screening instead of 830.

Figure 2 : Unclear what low risk of bias and high risk of bias are x axis is not labelled

Author’s response:

- A statement to this effect has been added to the methods section and the Figure 2 legend: “For each quality measure, fulfilling the criteria was interpreted as a score of 1 and ‘low risk of bias’.”
Figure 3 and 4 – while the bar plots of the numbers are useful for comparing the figures, perhaps stating the sample size below  the box plots on the  x axis and combining a swarm plot may help improve the clarity?

Author’s response:

Thanks for this suggestion – we have done this.

Figure 9: unclear what is being plotted here in the forest plot. In the text where Figure 9 is referenced, it is unclear what about the figure is being referenced.

Author’s response:

Sorry that this was not more clear. A figure legend has been added to improve the clarity of the figure: “Forest plot of the studies included in the final meta-regression model. Studies identified with the first author and year of publication. The log odds of mortality are shown along with 95% confidence intervals.”. The reference to the figure in the text has also been edited: “There was substantial heterogeneity (I^2 70.1%, df 264) between experimental groups, see the forest plot in Figure 9.”

Line 371- where are how is the data available? - This seems to be S9 but it is not cited in the text and the caption for S9 is missing

Author’s response:

- A reference to S9_Data has been added in the text.

Line 385- Perhaps the authors can clarify what is meant by unnecessary waste in this case

Author’s response:

We are grateful for this comment, and on reflection agree that this phraseology is somewhat subjective. The sentence has been edited to: “The number of mice per experimental group has become more consistent with time, as researchers are more aware and institutional guidance mandates reductions in experimental group sizes.”

Line 391- perhaps the authors can clarify what 'experiments of the kind' means here?

Author’s response:

On reflection, we deem this phrase unnecessary and it has been removed.

Lines 408-411-This reasoning suggests similarities in blood brain barrier development in mice and humans, but is this the only explanation for the contribution of mice age to heterogeneity? if yes, then such an explanation needs more support with data.

Author’s response:

The reviewer is right to point out that it may be over simplistic to consider the blood brain barrier similar in humans and mice without further evidence. Therefore, the sentence has been edited to: “Additional factors that are likely to play a role include the development of the immune system as well as maturation of the blood brain barrier; to what extent this is relevant to human disease is not clear, but in mice this is clearly relevant, with some investigators using a sham IC injection in order to disrupt the blood brain barrier and allow encephalitis to develop (18).”

Line 433- Minor point -Japanese encephalitis virus is not universally a Hazard group 3 pathogen

Author’s response:

Thanks for picking this up. ‘Hazard group 3 virus’ has been replaced by ‘dangerous pathogen’ and the potential risk to laboratory workers acknowledged (new line 512)

Figure S4- How was the phylogenetic tree made? How many sequences were included and excluded? How was genotype assigned?

- The S4 legend has been edited: “JEV phylogenetic tree including all complete genome sequences uploaded to GenBank (n=329) and partial sequences (n=4) if used in studies with sequences used in studies highlighted. This was created using MEGA-X and genotypes assigned manually.”
--------------------

Reviewer #1: Bharucha et al undertook extensive review of peer reviewed publications reporting mice as experimental animals for understanding Japanese Virus infection. This is, to the best of my knowledge, and as claimed by the authors, first such attempt to collate, review, analyse data on JEV mortality in mice. Publications from 1935 to 2020 are included. This is a commendable effort, conducted meticulously and is likely to be useful for further research since mice are still very commonly used animals to understand JE pathogenesis, for developing/testing drugs and vaccines. Authors encountered enormous diversity in published data in each of the parameters identified making their task of providing useful information for future work difficult. However, by decreasing heterogeneity within parameters and eliminating some parameters completely from the final analysis they could arrive at a reasonably sound and useful information, which is useful for future researchers.

Work is extremely useful for the scientists, technologists, drug and vaccine developers for JE, since mouse experiments are still needed for drug and vaccine development efforts.

Put in some nice comments thanking them for the positive reviews etc!

Some of the minor concerns are already highlighted. It will make the review more easily understandable to researchers who may not have extensive understanding of the statistical methods used.

Reviewer #2: Thank you for the opportunity to the review the manuscript titled “Mouse models of Japanese encephalitis virus infection: a systematic review and meta-analysis using a meta-regression approach” by Bharucha et al. The authors have analysed 127 separate studies which report mortality after inoculation of mice (total of 5026 animals) with virulent strains of Japanese encephalitis virus. They have performed meta regression analyses to identify factors that could account for the heterogeneity observed in the outcome (i.e. mortality). The authors hypothesized and subsequently showed that variations in - virus strain, dose and route of administration, mouse strain, age and sex contribute to observed heterogeneity in the reported mortality. Based on this they constructed a meta regression model with substantially reduced heterogeneity. This work clearly shows the value of such analysis to inform future experiments. In particular, for Japanese encephalitis virus infection
of mice, this study provides the framework for better experimental design and suggests reporting standards

This work is important and the study is clear, the data are convincing. All my concerns both conceptual and editorial are minor.

I have the following concerns regarding the study- given that none of the studies did particularly well on the quality score, how do the authors justify the use of data from such studies? Would setting a quality threshold impact heterogeneity? If not, then is this a relevant quality score?

Author’s response:

- The CAMARADES checklist is used to perform a combined assessment of the reporting of a number of measures to reduce bias, and several indicators of external validity and study quality. A histogram of the overall quality scores is shown below -

A sensitivity analysis was performed using different cut-offs (overall quality score ≥ 7,8,9,10,11,12,13) and this did not improve the heterogeneity of the final model. This is likely due to the fact that the score largely pertains to the quality of reporting. As we have described above, poor reporting does not necessarily indicate poor quality science, though there is not doubt that the ideal scenario is to have both the experimental work and the reporting done to a high standard. One of the purposes of our review is to point out that there is substantial heterogeneity which is not fully explained by the factors which we This has been elaborated in the methods section: “Sensitivity analysis was performed on the final model using subgroups with different overall quality scores.” and results: “A sensitivity analysis using subgroups with different thresholds for overall quality scores was performed and did not improve the heterogeneity.”

It would be good to have some background on why the specific factors were considered as the key variables  in the study  - are these kind of difference with respect to age and sex of mice for instance known for JEV? What other factors could be included but were not? For instance end point or duration of experiments is another variable, why was this not included in the study?

Author’s response:

The Key variables were chosen based fundamentally on biological plausibility, and also to some extent backed up by narrative reading of the literature and experience of other researchers in the field. Finally, the availability of enough data for an analysis was considered. The end point that we used was mouse death, as we considered that this was objective and consistently reported. Other factors, such as laboratory, were also considered in our analysis. Unfortunately, there were insufficient data reported to study alternative end points such as mouse behaviour or neurological presentations.

One would expect the amount of virus inoculated to correlate strongly with mortality, however, this is not clear from Figure 5. In the more homogeneous final model, does the equivalent of Figure 5 resolve this better?

Author’s response:

- As per your suggestion (previous comment), we have added a fitted line (and 95% CIs) to Figure 5b that shows the correlation of virus dose with mortality. Overall, increasing dose does correlate with increasing mortality. However, as we have shown, this nothing like captures all the variability in the literature, even when combined with other factors that plausibly affect the outcome. We have also attached to this resubmission the equivalent of Figure 5 with the filtered dataset used in the final model for your review (Figure 5_filtered); however this does not resolve the correlation better and if you agree, need not be included in the final manuscript.
--------------------

Figure Files:
While revising your submission, please upload your figure files to the Preflight Analysis and Conversion Engine (PACE) digital diagnostic tool, <https://pacev2.apexcovantage.com>. PACE helps ensure that figures meet PLOS requirements. To use PACE, you must first register as a user. Then, login and navigate to the UPLOAD tab, where you will find detailed instructions on how to use the tool. If you encounter any issues or have any questions when using PACE, please email us at [figures@plos.org](mailto:figures@plos.org).
